# Financial burdens of HIV and chronic disease on people living with HIV in Côte d’Ivoire: A cross-sectional out-of-pocket expenditure study

**DOI:** 10.1371/journal.pone.0255074

**Published:** 2021-07-29

**Authors:** Rachel D. Stelmach, Miriam Rabkin, Kouame Abo, Irma Ahoba, Mahena Gildas Anago, Rodrigo Boccanera, Hermann Brou, Rebecca Flueckiger, Kieran Hartsough, Martin Msukwa, Jennifer Zech, Felicity Young, Rachel Nugent

**Affiliations:** 1 RTI International, Washington, DC, United States of America; 2 ICAP at Columbia University, New York, NY, United States of America; 3 Programme National de Lutte Contre le SIDA (PNLS), Abidjan, Côte d’Ivoire; 4 Health Resources and Services Administration, Rockville, MD, United States of America; 5 RTI International, Atlanta, GA, United States of America; 6 RTI International, Brisbane, Australia; 7 RTI International, Seattle, WA, United States of America; University of Utah, UNITED STATES

## Abstract

**Background:**

Although people living with HIV in Côte d’Ivoire receive antiretroviral therapy (ART) at no cost, other out-of-pocket (OOP) spending related to health can still create a barrier to care.

**Methods:**

A convenience sample of 400 adults living with HIV for at least 1 year in Côte d’Ivoire completed a survey on their health spending for HIV and chronic non-communicable diseases (NCDs). In addition to descriptive statistics, we performed simple linear regression analyses with bootstrapped 95% confidence intervals.

**Findings:**

365 participants (91%) reported OOP spending for HIV care, with a median of $16/year (IQR 5–48). 34% of participants reported direct costs with a median of $2/year (IQR 1–41). No participants reported user fees for HIV services. 87% of participants reported indirect costs, with a median of $17/year (IQR 7–41). 102 participants (26%) reported at least 1 NCD. Of these, 80 (78%) reported OOP spending for NCD care, with a median of $50/year (IQR 6–107). 76 participants (95%) with both HIV and NCDs reported direct costs, and 48% reported paying user fees for NCD services. Participants had missed a median of 2 HIV appointments in the past year (IQR 2–3). Higher OOP costs were not associated with the number of HIV appointments missed. 21% of participants reported spending over 10% of household income on HIV and/or NCD care.

**Discussion and conclusions:**

Despite the availability of free ART, most participants reported OOP spending. OOP costs were much higher for participants with co-morbid NCDs.

## Introduction

Ensuring that people living with HIV know their status, receive treatment, and achieve viral suppression is critical for both individual and population-level outcomes [1]. Understanding and mitigating barriers to HIV testing, linkage to treatment, retention in care, and adherence to treatment is thus essential. Early in the epidemic, out-of-pocket (OOP) costs for HIV treatment were identified as substantial barriers to successful HIV treatment [2–4], and global donors and governments have committed to providing antiretroviral therapy (ART) free of charge in low-income countries [1]. However, free ART alone is often insufficient to prevent OOP spending, and in both high- and low-income settings, additional expenses such as user fees, pharmacy dispensing fees and travel costs have been shown to reduce adherence and retention [5, 6].

HIV epidemic control in West Africa has lagged behind other regions [7]. Although this lag stems from many factors, the region has some of the world’s highest OOP expenditures on health [8], and user fees are common in the public sector [9]. In Côte d’Ivoire, for example, the government provides free access to ART [10, 11], but charged user fees for HIV treatment until early 2019, when they were dropped in response to concerns about their impact on HIV testing, linkage and retention [11]. As in many other countries, people living with HIV pay for non-HIV health services and for indirect expenses such as transportation [12]. The rising prevalence of chronic non-communicable diseases (NCDs) makes this problematic, as people living with HIV are at higher risk of some NCDs than their HIV-negative peers, including cardiovascular disease, dyslipidemia, diabetes and cancer [13]. These OOP costs can jeopardize long-term disease management and reduce the quality of life for people who must spend household resources on medical care rather than other necessities or desires [14].

Côte d’Ivoire has a generalized HIV epidemic, with an adult prevalence of 2.6% and approximately 460,000 people living with HIV [15]. The national HIV program is supported by the Government of Côte d’Ivoire and its development partners, including the U.S. President’s Emergency Fund for AIDS Relief (PEPFAR) and the Global Fund for AIDS, TB and Malaria (Global Fund). UNAIDS estimates that among all people living with HIV in Côte d’Ivoire, 63% are aware of their status; of those aware of their status, 87% are on ART; and of those on ART, 75% are virally suppressed. That is, among all people living with HIV in Côte d’Ivoire, 55% are on ART, and 41% are virally suppressed [15].

NCDs are increasingly prevalent in Côte d’Ivoire, although population-based data are scarce [16]. Cardiovascular disease was the 5^th^ leading cause of death in 2017 [17], and a screening campaign the same year found HTN in 20% of 24,563 adults tested [18]. The International Diabetes Federation estimates that 2.4% of Ivorian adults have diabetes [19]. People living with HIV face a higher risk of these conditions—including cardiovascular disease, cervical cancer, depression and other mental health disorders, chronic kidney disease, and diabetes—than do people without HIV [13, 20–22]. Also, with ART, people living with HIV live to old age, which further increases their risk of NCDs [13, 23]. Due to potential efficiencies of combining care for multiple diseases, people with comorbidities often face costs different from would be expected by simply adding the costs of each of their diseases alone [23, 24]. To better understand the full picture of health care costs faced by people living with HIV, therefore, studies should include data on the costs of all conditions faced within a population.

We conducted a study of OOP spending in a convenience sample of people living with HIV in Côte d’Ivoire between March and May of 2019. We explored costs associated with HIV treatment as well as with chronic diseases. We explored linkages between OOP spending, missed care and measures of financial distress, including indicators of catastrophic expenditures. To our knowledge, this is the first study to examine the costs of both HIV and chronic disease care in Côte d’Ivoire.

## Methods

### Facilities

**Fig 1:**
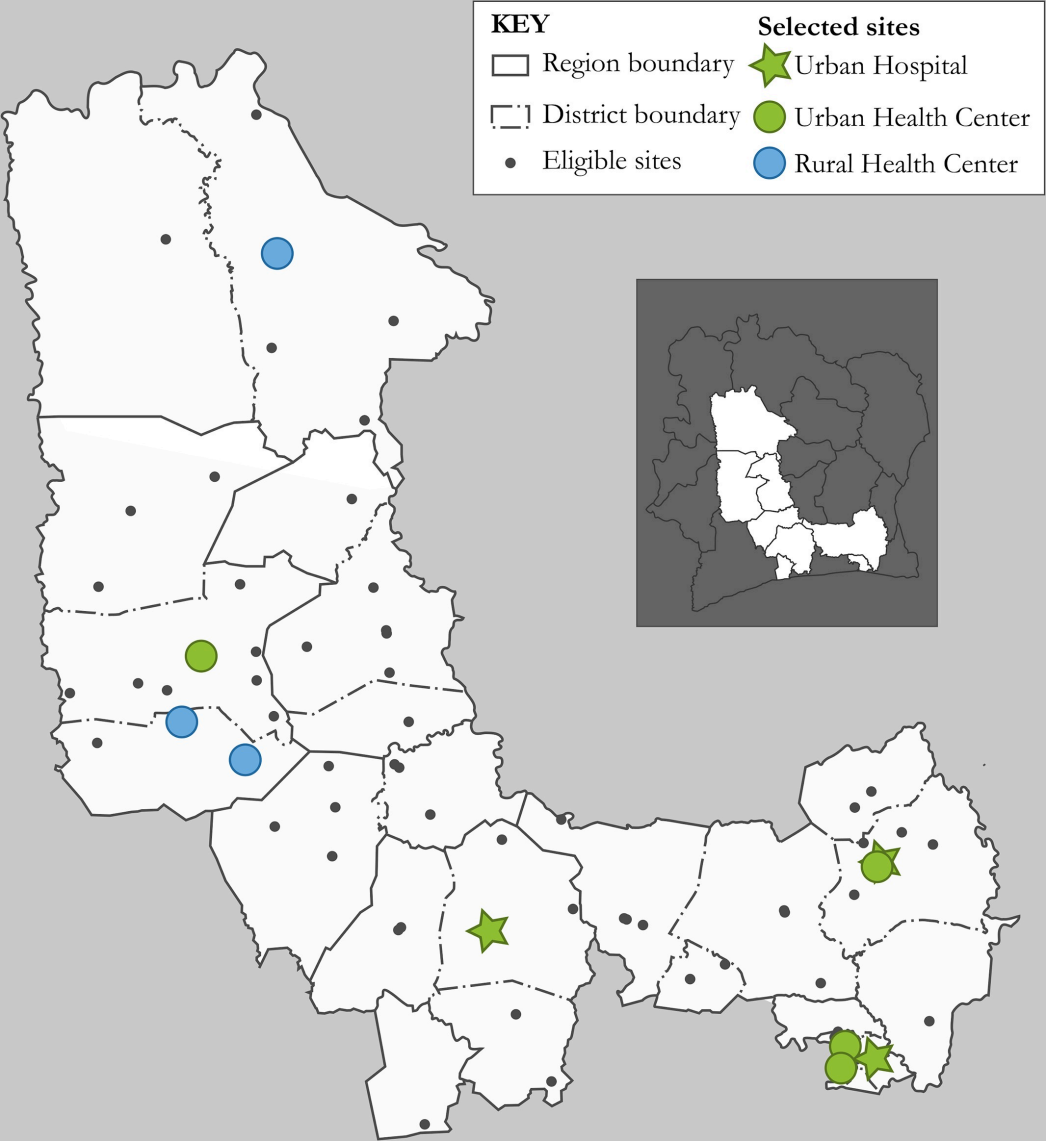
Map of included and eligible facilities in Côte d’Ivoire. (Figure created by the authors. Republished from [25] under a CC BY license, with permission from the International AIDS Society, original copyright 2020).

Nine health facilities were randomly selected from the 85 eligible PEPFAR-supported facilities with at least 100 patients on ART, stratified by type (hospital or health center) and location (urban or rural) (Fig 1, Table 1) [25]. As the random sample did not included any urban referral hospitals, one additional urban referral hospital was added to the sample.

**Table 1:** List of included facilities.

Facility	Region	Facility Type	Facility Location	Number of PLHIV on ART
Centre de Sante Urbain Com Angre	Abidjan 2	Health center	Urban	552
Complexe Socio-Urbain Com HKB de Blockauss	Abidjan 2	Health center	Urban	264
Hopital General de Bingerville	Abidjan 2	Hospital	Urban	935
Protection Maternelle Infantile Adzope	Agneby-Tiassa-Me	Health center	Urban	174
Hopital General de Adzope	Agneby-Tiassa-Me	Hospital	Urban	913
Centre AntiTuberculeux de Daloa	Haut-Sassandra	TB clinic/ Health center	Urban	328
Centre de Sante Urbain de Saioua	Haut-Sassandra	Health center	Rural	200
Centre de Sante Urbain de Boguedia	Haut-Sassandra	Health center	Rural	159
Centre de Sante Urbain de Sarhala	Worodougou-Bere	Health center	Rural	135
Centre Hospitalier Regional de Divo	Loh-Djiboua	Referral hospital	Urban	1363

### Participants

We recruited a convenience sample of HIV-positive adults scheduled for routine ART appointments at one of the study sites. Inclusion criteria included having missed at least one appointment in the previous 12 months; having been on ART for at least 12 months; speaking French, English, or a local language spoken by the interviewer; being at least 18 years old; and not being acutely ill on the day of the appointment. Recognizing that people who miss clinic appointments may be more likely to have financial barriers to attendance, we included people who were scheduled for an ART appointment but did not attend. The study clinics routinely call people who miss appointments; during these routine calls, potentially eligible patients were offered the opportunity to participate either by coming to the health facility later that week, or by providing information via phone. Because chronic NCDs occur more frequently among older people, we purposively recruited a sample with an equal number of participants aged 18–39 years old and 40 or more years old.

### Data collection

We developed a 100-question survey which was written in English, translated into French and then back-translated to English and pre-tested to ensure accuracy [see S1, S2 Files]. The survey was piloted with 30 HIV-positive adults at two health facilities in Cote d’Ivoire; no substantive changes were required as a result. Study staff fluent in local languages that are typically oral not written (*e*.*g*., Senoufo and Dioulain) also practiced questions and clarifications in those languages. The survey included questions about out of pocket costs for health services related to HIV and/or other chronic illnesses including: “high blood pressure or hypertension,” “high blood sugar or diabetes,” “heart disease or a chronic heart condition,” “lung disease or chronic lung condition,” “cancer or a tumor,” “depression” and “any other chronic disease or disease that is long lasting.” It was programmed onto tablets and administered by trained interviewers in French, English, Senoufo and/or Dioulain in person or over the phone; it took between 30 and 45 minutes to complete. Participants were compensated the equivalent of $10 for their time, and those interviewed at a health facility received refreshments such as fruit juice or cookies.

Data were gathered on encrypted tablets and automatically uploaded for storage on SurveyCTO, a cloud-based system, and transferred for data analysis through DatAnywhere, a secure encrypted file transfer protocol. Data were exported to Microsoft Excel for Office 365 and analyzed using R (version 3.5.1) through RStudio (version 1.1.456). All costs were recorded in 2019 Cote d’Ivoire francs and converted to 2019 USD using the median conversion rate during data collection, 584 CFA = 1 USD [26].

### Data analysis

The target sample size was 400 participants, which is in line with similar studies of OOP expenditures for people living with HIV in sub-Saharan African and allows for descriptive statistics of the participants’ OOP expenditures [27, 28]. We conducted descriptive statistics using median, range, and interquartile range (IQR) to describe results. Where data were missing for a particular observation of a variable, we dropped that observation from the analysis of that variable. We also conducted simple tests of significance to explore the associations between costs of HIV care and total costs of HIV and chronic disease care with the number of HIV appointments missed. For both, we performed simple linear regressions with bootstrapped 95% confidence intervals (CIs) using the bias-corrected and accelerated method. This method of CI generation corrects for non-normal distributions, which is important given the often-skewed nature of cost data [29]. To examine affordability of care, we compared the total OOP expenditure as a percentage of the median value of the reported household income in each quintile. We then compared these percentages to the WHO Sustainable Development Goal out of pocket expenditure thresholds of 10% and 25% of household incomes [30].

### Ethical review

The study was approved by the Columbia University Medical Center IRB (#AAAS171), the Côte d’Ivoire Comité National d’Ethique des Sciences de la Vie et de la Santé (#182-18/MSHP/CNESVS-kp), the Côte d’Ivoire Ministry of Health, and the U.S. Health Resources and Services Administration (HRSA). All participants provided informed consent.

### Role of the funding source

The study sponsor had no role in the design of the study; the collection, analysis, or interpretation of the data; the writing of the report; or the decision to submit for publication.

## Results

### Description of participants

Between 19 March and 28 May 2019, 400 people participated in the survey. Of these, 376 (94.0%) were interviewed in person at the facility, and 24 (6.0%) were interviewed by phone. Table 2 summarizes participant characteristics by facility type and location.

**Table 2:** Overview of participants by facility type and location.

	Rural health center	Urban health center	Urban hospital	All
N	77	135	188	400
Age	42 (IQR: 35–50; range: 18–70)	37 (IQR: 32–44; range: 22–69)	41 (IQR: 35–49; range: 18–69)	39 (IQR: 33–49; range: 18–70)
Female	53 (68.8%)	110 (81.5%)	146 (77.7%)	309 (77.2%)
Married or living with partner	48 (62.3%)	67 (49.6%)	103 (54.8%)	218 (54.5%)
Number of people in household	4 (IQR: 3–7; range: 1–20)	4 (IQR: 3–6; range: 1–20)	5 (IQR: 3–7; range: 1–15)	5 (IQR: 3–7; range: 1–20)
Number of people older than 18 in household	2 (IQR: 2–4; range: 1–8)	2 (IQR: 2–4; range: 1–12)	2 (IQR: 2–4; range: 1–12)	2 (IQR: 2–4; range: 1–12)
Has health insurance	4 (5.2%)	13 (9.7%)	13 (6.9%)	30 (7.5%)
HIV appointments attended per year	8 (IQR: 5–10; range: 2–12)	9 (IQR: 5–11; range: 1–12)	6 (IQR: 4–10; range: 1–13)	8 (IQR: 4–10; range: 1–13)
Months on ART	60 (IQR: 36–86; range: 12–245)	49 (IQR: 28–84; range: 12–276)	59 (IQR: 36–96; range: 12–168)	56 (IQR: 33–89; range: 12–276)
**Education level**				
Higher/more than secondary	0 (0.0%)	23 (17.0%)	7 (3.7%)	30 (7.5%)
Secondary	15 (19.5%)	39 (28.9%)	57 (30.3%)	111 (27.8%)
Primary	38 (49.4%)	36 (26.7%)	66 (35.1%)	140 (35.0%)
None	24 (31.2%)	37 (27.4%)	58 (30.9%)	119 (29.8%)
**Occupation**				
Self-owned business	21 (27.3%)	50 (37.0%)	90 (47.9%)	161 (40.2%)
Farmer	38 (49.4%)	10 (7.4%)	30 (16.0%)	78 (19.5%)
None	8 (10.4%)	27 (20.0%)	18 (9.6%)	53 (13.2%)
Other	10 (13.0%)	48 (35.6%)	50 (26.6%)	108 (27.0%)
**Personal monthly income (2019 USD)**				
$513-$1,026	1 (1.3%)	3 (2.2%)	5 (2.7%)	9 (2.2%)
$205-$513	2 (2.6%)	16 (11.9%)	16 (8.5%)	34 (8.5%)
$103-$205	9 (11.7%)	22 (16.3%)	20 (10.6%)	51 (12.8%)
$55-$103	19 (24.7%)	27 (20.0%)	33 (17.6%)	79 (19.8%)
$38-$55	17 (22.1%)	23 (17.0%)	28 (14.9%)	68 (17.0%)
<$38	29 (37.7%)	39 (28.9%)	80 (42.6%)	148 (37.0%)
Unknown	0 (0.0%)	5 (3.7%)	5 (2.7%)	10 (2.5%)
Refuses to answer	0 (0.0%)	0 (0.0%)	1 (0.5%)	1 (0.2%)

### Costs of HIV care

#### Overall OOP costs (direct and indirect)

**Fig 2:**
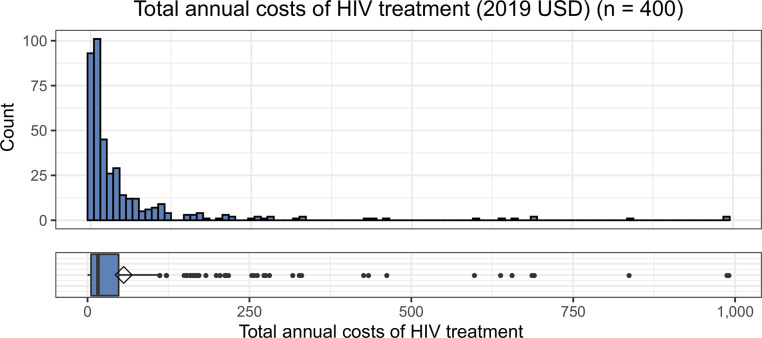
Total annual costs of HIV treatment (2019 USD) (n = 400).

As shown in Table 3, 365 participants (91.3%) reported OOP expenditures related to their HIV care; 35 (8.8%) reported paying no HIV-related OOP costs. Participants reported a median total OOP expenditure related to their HIV care, including both direct and indirect costs, of 16 USD (IQR: 5–48) per year. Direct costs include money spent on the goods or services themselves, such as payments for medication, tests, or hospitalizations. Indirect costs include other resources lost due to the patient’s receiving the services, such as payments for transportation, wages lost, or compensation given in exchange for child care. Excluding inpatient hospitalization costs, participants reported a median total cost of 14 USD (IQR: 5–43) per year. Among those who reported any HIV-related OOP costs, the median reported was 21 USD (IQR: 7–56) per year. These costs are highly right-skewed; that is, most participants reported relatively low costs of HIV care, but several outliers reported higher costs (Fig 2).

**Table 3:** Annual costs of HIV care (N = 400) (2019 USD).

			All participants					Only participants reporting a cost within the category				
	n	%	Med	Q1	Q3	Min	Max	Med	Q1	Q3	Min	Max
Total	365	91.3%	16	5	48	0	992	21	7	56	0	992
Direct costs	136	34.0%	0	0	1	0	657	2	1	41	0	657
*Medication*	*32*	*8*.*0%*	*0*	*0*	*0*	*0*	*626*	*70*	*25*	*124*	*2*	*626*
*Tests*	*64*	*16*.*0%*	*0*	*0*	*0*	*0*	*226*	*3*	*1*	*5*	*1*	*226*
*Hospitalization*	*19*	*4*.*8%*	*0*	*0*	*0*	*0*	*428*	*63*	*43*	*163*	*21*	*428*
*Other (gloves)*	*49*	*12*.*2%*	*0*	*0*	*0*	*0*	*21*	*1*	*1*	*1*	*0*	*21*
Indirect costs	349	87.2%	12	5	38	0	990	17	7	41	0	990
*Transportation*	*342*	*85*.*5%*	*8*	*3*	*21*	*0*	*216*	*10*	*6*	*24*	*0*	*216*
*Lost wages*	*81*	*20*.*2%*	*0*	*0*	*0*	*0*	*941*	*38*	*21*	*86*	*3*	*941*
*Child care*	*10*	*2*.*5%*	*0*	*0*	*0*	*0*	*821*	*26*	*12*	*201*	*5*	*821*

n = number reporting any costs within the category; % = percent of N reporting a cost within the category; med = median; Q1 = 25^th^ percentile; Q3 = 75^th^ percentile; min = minimum; max = maximum. Values within each row show the distribution of costs within that category either across all participants or within only those participants with a non-zero cost within that category; as different numbers of people reported values for each row, the values for subcategories are very unlikely to add up to a category’s total.

#### Direct OOP costs

136 participants (34.0%) reported paying direct costs for HIV care, such as payments for medication, tests, hospitalization and/or gloves. No participants reported paying for ART or paying a user fee at their most recent visit. Those reporting direct costs paid a median of 2 USD (IQR: 1–41) in direct OOP expenditures per year.

#### Indirect OOP costs

349 participants (87.2%) reported indirect costs, primarily payment for transportation and lost wages. Participants reporting any indirect costs paid a median of 17 USD (IQR: 7–41) per year. 178 participants paid only transportation costs, which represents 48.8% of participants reporting any OOP costs.

#### Costs by covariates

Median reported annual costs differed by location and facility type. The 135 participants (33.8%) who received care from urban health centers reported the highest median costs of 21 USD per year (IQR 7–62, range 0–989), followed by the 188 participants (47.0%) from urban hospitals at 18 USD per year (IQR 5–62, range 0–992), and the 77 participants (19.2%) from rural health centers at 9 USD per year (IQR 3–23, range 0–688). Direct costs did not differ markedly by location; most of the differences in total costs arose from differences in indirect costs. In particular, participants from urban health centers reported higher median transportation costs than did participants from urban hospitals or rural health centers.

**Fig 3:**
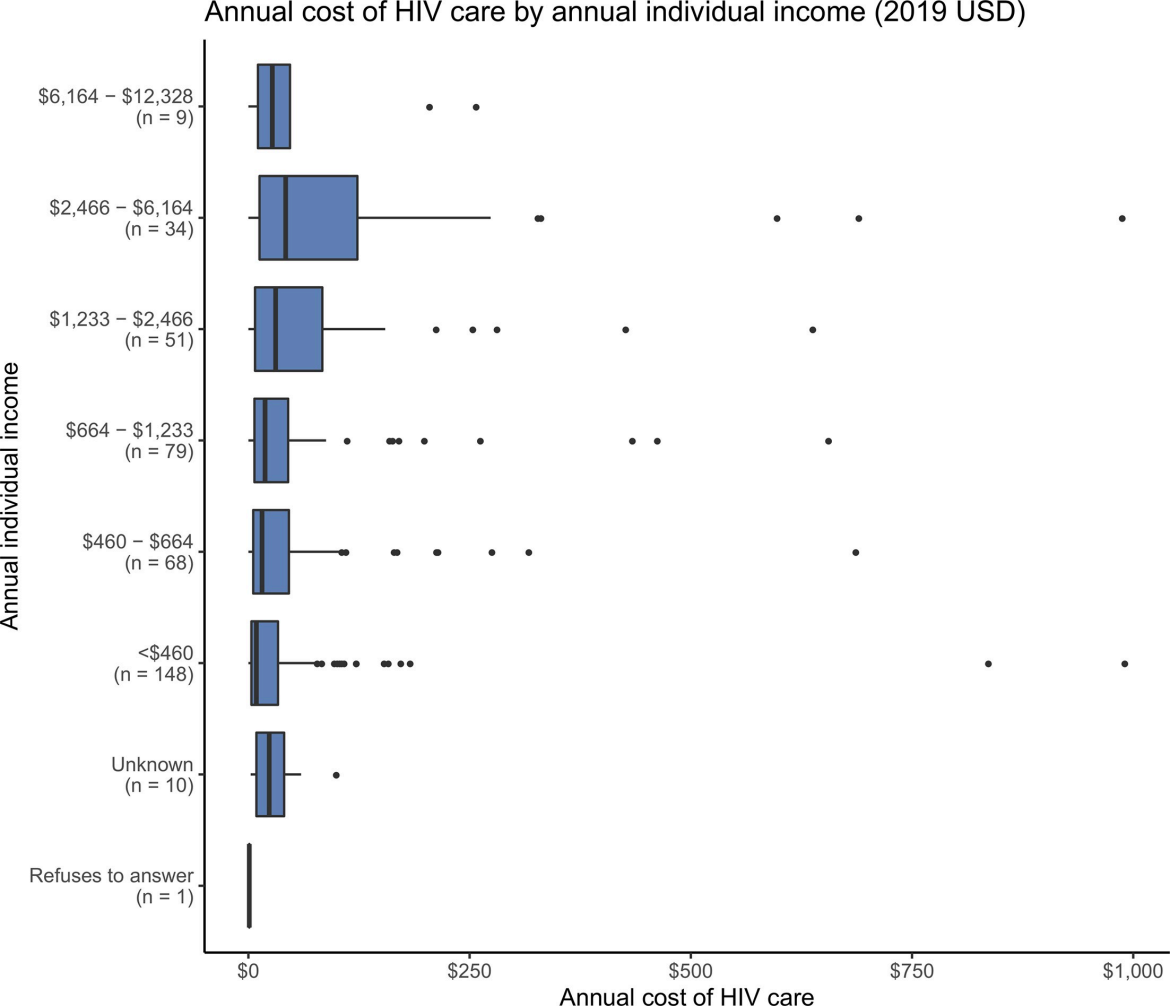
Annual cost of HIV care by annual individual income (2019 USD) (n = 400).

Men reported higher median annual HIV costs (27 USD per year, IQR 7–66, range 0–989 USD) than did women (15 USD per year, IQR 5–43, range 0–992). Participants with higher individual incomes generally reported higher median costs (Fig 3), though all income bands included high outliers. For additional context relating income to costs, please see the below section on affordability. Median costs of HIV treatment were very similar among those with and without health insurance, among those of different ages, and among those with different lengths of time on ART.

### Missing HIV appointments and ART refills

As required by the inclusion criteria, all participants had missed at least one appointment in the past year; overall, participants missed a median of two appointments in the previous year (IQR: 2–3). The most common reasons for missing appointments were that the participant was travelling or away from home [141 participants (35.2%)]; was unable to leave work, school, or home [102 participants (25.5%)]; forgot their appointment [82 participants (20.5%)]; or had a problem with their transportation [51 participants (12.8%)]. Of note, only 27 participants (6.8%) cited OOP cost as a reason for missed appointments and only 21 (5.3%) cited cost as their *primary* reason for missing an appointment. Within this sample, higher OOP costs for HIV treatment do not appear to be associated with the number of missed HIV appointments in a simple linear regression (for overall costs, 95% CI -0.0004 to 0.0043).

None of the respondents reported OOP payments for ART, but some did report paying for medications related to HIV, such as medications for opportunistic infection prevention or treatment. Of the 33 participants (8.25%) who reported paying for HIV-related medication in the past 12 months, 11 (33.3%) had missed at least one refill of that medication in the past month. Among those who reported paying for medication, those who missed a refill reported higher median annual costs of medication [125 USD (IQR: 87–174)] than did those who did not miss a refill [38 USD (IQR: 13–72)]. Of the 11 people who gave reasons for skipping refills of medication related to HIV, 9 (81.8%) said it was because the medication was too expensive and/or they could not afford it.

### Costs of chronic disease care

102 participants (25.5% of the total) reported at least one chronic illness. Of these 102 participants, 85 (83.3%) reported one chronic illness, 13 (12.7%) reported two, 2 (2.0%) reported three, and two (2.0%) reported four. Sixty-one participants (48.8% of the 102 reporting a chronic illness) reported at least one “classic” NCD, *i*.*e*., hypertension, cardiovascular disease, diabetes, chronic lung disease, and/or cancer. Participants also reported other chronic or recurring conditions: gastric ulcers, genital disease, blood disease, skin disease, sinusitis, renal disease, joint disease, hypotension, ear disease, umbilical hernia, nerve disease, liver disease, hemorrhoids, headaches, fatigue, and diarrhea.

The most common chronic illnesses reported were hypertension (28 people, 7.0% of the 400 participants) and lung disease (25 people, 6.3%). Stomach ulcers were the third most common, with 20 (5.0%) occurrences.

#### Overall OOP costs (direct and indirect)

Table 4 details the OOP costs related to chronic disease care. Of the 102 people with a chronic illness, 80 (78.4%) reported related OOP expenditures. The median OOP cost of chronic disease care for all 102 participants reporting a chronic disease was 50 USD (IQR: 6–107); among the 80 people paying OOP for chronic disease care, the median annual expenditure was 80 USD (IQR: 32–127).

**Table 4:** Annual costs related to chronic disease care (N = 102) (2019 USD).

			All participants with a non-HIV chronic disease					Only participants reporting a cost within the category				
	n	%	Med	Q1	Q3	Min	Max	Med	Q1	Q3	Min	Max
Total	80	78.4%	50	6	107	0	63,210	80	32	127	3	63,210
Direct costs	76	74.5%	38	0	95	0	62,183	72	26	115	2	62,183
*Medication and tests*	*63*	*61*.*8%*	*26*	*0*	*86*	*0*	*61*,*875*	*75*	*36*	*107*	*3*	*61*,*875*
*Facility user fees*	*49*	*48*.*0%*	*0*	*0*	*7*	*0*	*308*	*7*	*3*	*21*	*2*	*308*
*Hospitalization*	*2*	*2*.*0%*	*0*	*0*	*0*	*0*	*77*	*54*	*42*	*65*	*31*	*77*
Indirect costs	59	57.8%	2	0	10	0	2,669	8	4	21	0	2,669
*Transportation*	*57*	*55*.*9%*	*1*	*0*	*7*	*0*	*205*	*6*	*2*	*12*	*0*	*205*
*Lost wages*	*15*	*14*.*7%*	*0*	*0*	*0*	*0*	*2*,*669*	*10*	*5*	*36*	*1*	*2*,*669*
*Child care*	*2*	*2*.*0%*	*0*	*0*	*0*	*0*	*821*	*421*	*221*	*621*	*21*	*821*

n = number reporting any costs within the category; % = percent of N reporting a cost within the category; med = median; Q1 = 25th percentile; Q3 = 75th percentile; min = minimum; max = maximum. Values within each row show the distribution of costs within that category either across all participants or within only those participants with a non-zero cost within that category; as different numbers of people reported values for each row, the values for subcategories are very unlikely to add up to a category’s total.

#### Direct OOP costs

Of the 80 people who reported any cost for chronic disease care, 76 (95.0%) reported direct OOP costs, with a median of 72 USD (IQR: 26–115). In contrast to HIV care, 49 participants (48%) reported paying user fees for chronic disease related care. Among those who paid user fees, the median user fee was 3 USD (IQR: 2–4) per appointment, and the median number of appointments was 1 per year (IQR: 1–10), which led to a median annual cost of user fees of 7 USD (IQR: 3–21).

#### Indirect OOP costs

Of the 80 people who reported any cost for chronic disease care, 59 (73.8%) reported indirect costs. As with HIV care, the most common indirect cost reported for chronic disease care was transportation, with 57 participants (71.3%) paying for chronic disease care reporting OOP transportation costs. Participants reporting any indirect costs paid a median of 8 USD (IQR: 4–21) per year.

#### Costs by covariates

Of the 66 participants receiving treatment for at least one chronic disease, 53 (80.3%) sought their HIV care in an urban location, and 13 (19.7%) sought their HIV care in a rural location. As with HIV costs, participants who sought HIV care in urban locations also reported higher median costs of non-HIV chronic disease care (53 USD, IQR 7–125 USD, range 0–63,210) than did those who sought HIV care in rural locations (32 USD, IQR 0–75 USD, range 0–313). Although median indirect costs were slightly higher in urban areas, the largest difference occurred in the direct costs of non-HIV chronic disease care, with urban participants reporting a median of 40 USD in direct costs (IQR 3–102 USD, range 0–73) and rural participants reporting a median of 31 USD (IQR 0–73, range 0–62,183). All subcategories of direct costs showed a higher median cost in urban and rural areas.

Unlike with HIV, female participants reported higher median annual costs for non-HIV chronic disease (54 USD, IQR 11–106, range 0–479) than did male participants (27 USD, IQR 0–109, range 0–63,209). These differences arose primarily from differences in direct costs, including the fact that both participants who reported direct costs from hospitalization due to a non-HIV chronic disease were female.

Also unlike HIV, among the 102 participants with at least one non-HIV chronic disease, the 6 (5.9%) participants with health insurance reported lower median costs of non-HIV chronic disease care (3 USD, IQR 0–12, range 0–63,210) than did the 96 (94%) without health insurance (median 53 USD, IQR 8–108, range 0–2,913). The two insurance groups reported similar indirect costs, with the difference deriving primarily from higher direct costs incurred by those without insurance. Annual median total costs of non-HIV chronic disease treatment did not differ very much between age groups or income brackets. Please see the below section on affordability for a comparison of overall non-HIV chronic disease treatment with household income.

### Chronic disease costs and missed care

Only 18 people reported missing chronic disease appointments, which limits the ability to make comparisons within the group. The 18 respondents who reported missing a chronic disease appointment missed a median of 3 appointments per year (IQR: 2–4).

Of the 33 people who gave reasons for skipping chronic disease medication refills, 32 (97.0%) said it was because the medication was too expensive and/or they could not afford it. Two people, including one who also cited cost in their reasons for skipping the medication, said that they skipped the medication refill because they were ill or unable to travel because of their symptoms.

When added together, the median annual cost of HIV care and chronic disease care combined for all participants was 106 USD per year (IQR: 39–164; range: 0–64,198). Importantly, these costs might not reflect a person’s or household’s total medical expenses; they do not capture the costs of other illnesses the patient might have, nor do they capture the medical expenses of other members of their household. Within this sample, higher costs related to HIV and chronic disease treatment combined do not appear to be associated with missing HIV appointments in a simple linear regression (95% CI -0.0004 to 0.0006).

### Indicators of financial distress

**Fig 4:**
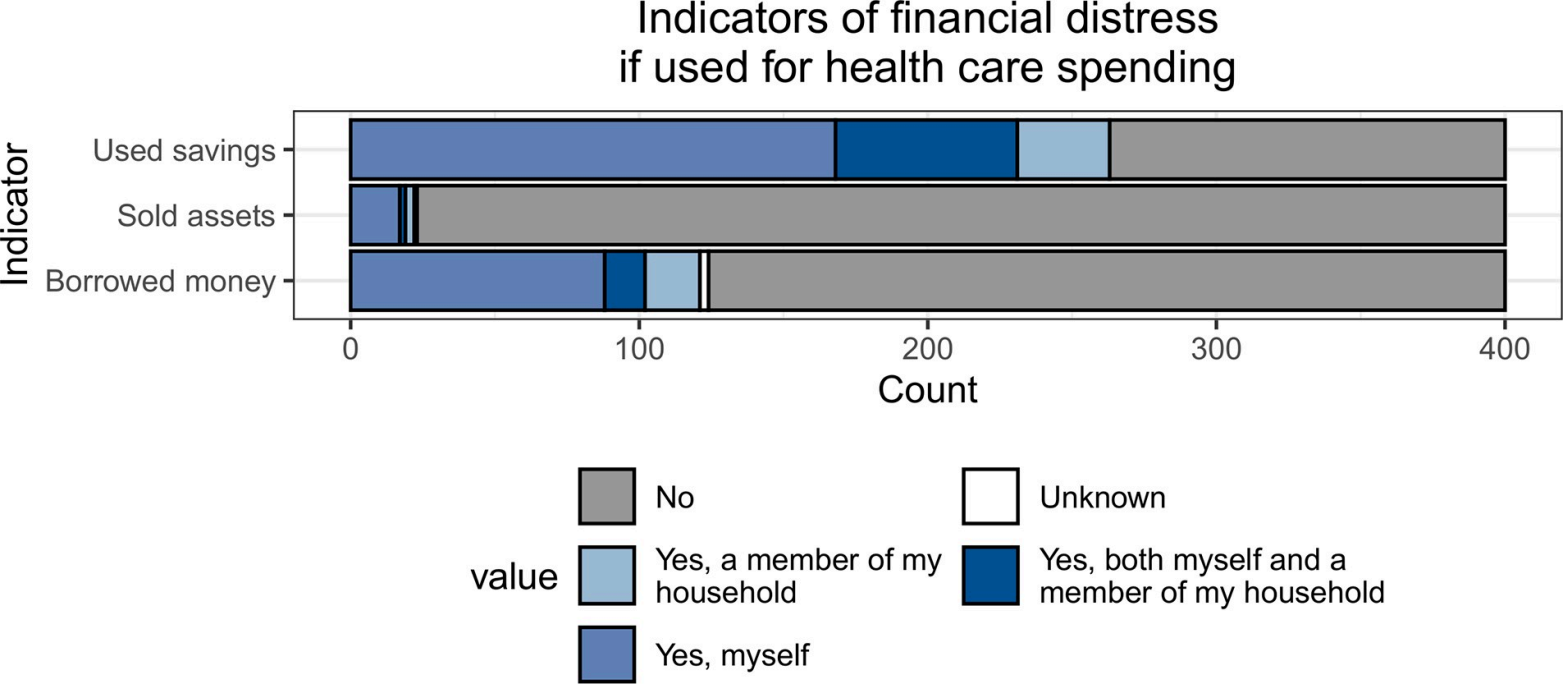
Indicators of financial distress if used for health care spending (n = 400).

The distribution of participants who used savings, borrowed money, or sold assets to pay for health care appears in Fig 4. Two-thirds of participants—263 (65.8%)—reported that they or someone in their household had used savings to pay for health care costs and 121 (30.2%) reported that they or someone in their household had borrowed money to pay for health care costs. Relatively few—22 (5.5%)—had sold assets to pay for health care. Results were similar whether the money was spent related to HIV services, chronic disease services, or both.

In general, people who reported an indicator of financial distress within the past year reported higher median costs for HIV and/or chronic disease treatment. Furthermore, people who reported a chronic disease and an indicator of financial distress reported higher total costs of HIV and/or chronic disease treatment than those who did not report a chronic disease (Table 5).

**Table 5:** Total costs of HIV and chronic disease care by indicators of financial distress (N = 400) (2019 USD).

	Has a chronic disease	n	%	Med	Q1	Q3	Min	Max
**Used savings**								
Yes	Yes	74	18.5%	121	53	190	4	64,198
Yes	No	189	47.2%	19	6	64	0	992
No	Yes	28	7.0%	35	20	92	0	463
No	No	109	27.2%	8	5	31	0	599
**Sold assets**								
Yes	Yes	10	2.5%	141	90	187	4	721
Yes	No	12	3.0%	26	7	72	2	110
No	Yes	92	23.0%	103	37	157	0	64,198
No	No	285	71.2%	14	5	43	0	992
Unknown	No	1	0.3%	112	112	112	112	112
**Borrowed money**								
Yes	Yes	44	11.0%	135	80	194	4	886
Yes	No	77	19.2%	23	9	74	0	332
No	Yes	57	14.2%	78	26	138	0	64,198
No	No	219	54.8%	10	4	41	0	992
Unknown	Yes	1	0.3%	29	29	29	29	29
Unknown	No	2	0.5%	423	215	630	8	838

*n = number in category; Med = median; Q1 = 25th percentile; Q3 = 75th percentile; min = minimum; max = maximum

### Affordability

At the household level, participants reported spending a median of 1.7% of household income on HIV care (IQR: 0.4%-5.5%; range: 0%-431%). 59 participants reported spending more than 10% of their household income on their HIV care, and 21 participants reported spending more than 25% of their household income on their HIV care. Two participants reported spending more than their household income each year on their HIV care. These two participants both reported using their savings to finance health care expenditures, and one was unsure if their household had borrowed money to finance health care.

Including both chronic disease and HIV care, participants reported spending a median of 2.6% of their annual household income on chronic disease and/or HIV care (IQR: 0.5%-8.2%; range: 0.0%-1,490%). 85 participants reported spending more than 10% of their household income on chronic disease and/or HIV care, and 36 reported spending more than 25%. Six participants reported spending more than 100% of their household income on health care. All 6 of these participants reported using savings to finance their health care, and 1 reported borrowing money.

## Discussion and conclusions

In this purposive sample of 400 people on ART in Côte d’Ivoire, 91% of participants reported OOP costs related to their HIV care, with a median annual OOP expenditure of 16 USD (IQR: 5–48) for outpatient HIV services. The majority of this OOP spending was for indirect costs such as transportation, lost wages and child care; although 34% of participants reported direct costs, the median amount of direct costs was 2 USD (IQR: 1–41) per year. The most common OOP cost was transportation, which has previously been found to drive costs of HIV care and treatment in Côte d’Ivoire and has been noted as a barrier to ART treatment in sub-Saharan Africa for over a decade [12, 31]. The relatively high levels of indirect costs show even a program that covered all of the direct costs of HIV treatment might still leave HIV treatment unaffordable to some who need it. Providing free or subsidized transportation to HIV care would reduce these, though such a program would have to be careful not to unintentionally “out” people’s HIV status to their community and not to increase any stigma around seeking HIV care.

The vast majority of literature on the costs of ART in sub-Saharan Africa focuses on the costs to providers, rather than the out-of-pocket costs to patients. Several studies of OOP costs have been conducted, however. A 2019 study in Nigeria found OOP costs for ART similar to that in this study, with mean annual OOP spending of $21.76 USD, including both direct and indirect costs [32]. Similarly, a 2018 study in Tanzania found a mean annual OOP expenditure on HIV treatment of $40.37 for males and $28.01 for females, excluding costs of ART and user fees [26]. As in our study, transportation was often the largest portion of costs. Another study conducted in Nigeria in 2018, however, found much higher OOP costs for people living with HIV, at an average cost of $528 per year, including user fees and ART [27]. A 2015 study in KwaZulu-Natal, South Africa found much higher OOP costs than found in this study for people accessing ART and/or tuberculosis care: $412 per year in 2010 USD, excluding user fees [5].

These findings suggest that the Government of Cote d’Ivoire has effectively limited the financial impact of HIV care and treatment for people living with HIV. However, as the population of people on ART ages over time, the financial burden of their chronic NCDs is likely to have increasing impact [33]. We found that 25% of respondents reported at least one chronic NCD, and that OOP costs for these illnesses were substantially higher than for HIV, with a median annual OOP expenditure of 50 USD (IQR: 6–107). In contrast to their spending on HIV, participants incurred substantial direct as well as indirect OOP costs, and 48% reported paying facility user fees. To our knowledge, this is the first study to assess the costs of all comorbid chronic diseases borne by a sample of people living with HIV in Sub-Saharan Africa. Investing in NCD and risk factor screening, prevention, and treatment for people on ART is likely to improve their long-term health and protect them from added financial distress [34, 35].

Few participants reported costs as a perceived barrier to their HIV care, and neither the cost of HIV services nor the combined cost of HIV and chronic disease care were associated with the number of HIV appointments missed. However, 85 participants (21.3%) reported spending more than 10% of their household income on chronic disease and/or HIV care, two-thirds of participants reported that they or someone in their household had used savings to pay for health care costs in the past year, and almost one-third reported that they or someone in their household had borrowed money to pay for health care in the past year. These indicators of financial distress due to medical costs could reflect costs to the participant for conditions other than HIV or chronic illness, or they could reflect the costs of medical care for other members of the household. In either case, they show that policy makers must consider that people living with HIV must also deal with other health care-related conditions; policies that address HIV alone will not solve all the problems faced by people living with HIV.

Although this study provides a detailed look at the HIV and chronic illness-related OOP expenditures within our sample, it has several limitations. The purposive nature of the sample limits the generalizability of the findings, and the sample size precluded some statistical tests of significance. Furthermore, to ensure that all our participants could provide some data on barriers to ART, we limited the sample to only people who had missed at least one ART appointment in the previous 12 months. This restriction further limits the generalizability of the findings, though we expect that any reported barriers to care would be less important for those who had not missed any ART appointments in the previous 12 months. In addition, the indicators of financial distress refer to all health care spending, not just the costs captured in this survey. As we only solicited income bracket information rather than specific income data, the percentage-based measures of catastrophic health care spending are estimates. And finally, the survey asked people to report their treatments and costs from the past year, which could lead to recall bias. We mitigated recall bias for the cost calculations by asking patients about their costs for their most recent visit and then multiplying that cost by the number of reported treatments for the past year, but this could have introduced additional error if the most recent visit was more or less expensive than usual.

Despite these limitations, this detailed picture of the HIV and chronic illness OOP expenditure in Côte d’Ivoire provides important context for decisions about funding, policies, and program design. By better understanding the costs borne by people living with HIV, the government of Côte d’Ivoire and its partners can better craft policies to reduce the negative economic effects of HIV and chronic illness on its population.

## Supporting information

S1 File Questionnaire (English). (DOCX)

S2 File Questionnaire (French). (DOCX)
