# Supplementary material for: Financial burdens of HIV and chronic disease on people living with HIV in Côte d’Ivoire: A cross-sectional out-of-pocket expenditure study
Source: PLoS One. 2021 Jul 29;16(7):e0255074. doi: 10.1371/journal.pone.0255074 (PMC8320983; doi:10.1371/journal.pone.0255074)
Supplement: S1 File — (DOCX) [file pone.0255074.s001.docx]

| 1. Facility OpCon code   *To be completed by interviewer* |  |
| --- | --- |
| 1. Participant OpCon number   *To be completed by interviewer* |  |
| 1. How is the participant being interviewed?   *To be completed by interviewer* | - In person at the facility (1) - By phone (2) |
| 1. What is the participant’s sex?   *To be completed by interviewer* | - Male (1) - Female (2) - Unknown (99) |
| 1. How old were you on your last birthday? | - _______________________________________ [years] - Unknown/Refuses to answer (99) |
| 1. What is the highest level of school you have attended, if any? | - None (1) - Primary (2) - Secondary (3) - Higher/more than secondary (4) - Refuses to answer (99) |
| 1. What is your marital status? | - Married or living together (1) - Divorced/separated (2) - Widowed (3) - Never married or never lived together (4) - Refuses to answer (99) |
| 1. What is your primary occupation, that is, what kind of work do you mainly do?   *Interviewer to read list* | - Farmer (1) - Professional in Public Sector (2) - Professional in Private Sector (3) - Self-owned business (e.g., merchant) (4) - Other business (e.g., works in a shop) (5) - Services (e.g., maid, gardener) (6) - Student (7) - Sex worker (8) - None (9) - Other, *please specify* (10) - Refuses to answer (99) |
| 1. How much money did you earn last month?   *Please include income from employment (wages, salaries, tips, etc.), income from investments, income from pensions, regular case income, and income from government or social assistance. Do not include income from one-time receipts, such as inheritance or insurance settlement, or the value of goods produced for barter or own consumption.* | - < 22,393 francs (1) - 22,394-32,340 francs (2) - 32,341-60,000 francs (3) - 60,001-120,000 francs (4) - 120,001-300,000 francs(5) - 300,001-600,000 francs (6) - > 600,000 francs (7) - Unknown (88) - Refuse to answer (99) |
| 1. How many persons currently live in your household? | - _______________________________________ - Unknown (88) - Refuses to answer (99) |
| 10a. How many persons over the age of 18 currently live in your household? | - _______________________________________ - Unknown (88) - Refuses to answer (99) |
| 1. How much money did your household earn over the past 12 months?   *Please estimate to the best of your ability.*  *Please include income from employment (wages, salaries, tips, etc.), income from investments, income from pensions, regular case income, and income from government or social assistance. Do not include income from one-time receipts, such as inheritance or insurance settlement, or the value of goods produced for barter or own consumption.* | - < 268,716 francs (1) - 268,717-388,080 francs(2) - 388,081-720,000 francs (3) - 720,001-1,440,000 francs(4) - 1,440,001-3,600,000 francs (5) - 3,600,001-7,200,000 (6) - > 7,200,000 (7) - Unknown (88) - Refuses to answer (99) |
| 1. Do you have health insurance? | - Yes, National Health Insurance (1) - Yes, Community Health Insurance (2) - Yes, Private Insurance (3) - No (4) - Unknown (88) - Refuses to answer (99) |
| 1. How often have you come to a facility for HIV care and treatment appointments over the past 12 months? *Please include today’s visit in the total.*   FOR MISSED APPOINTMENT PARTICIPANTS: How often have you come to (*name the facility*) for HIV care and treatment appointments over the past 12 months? | - _________________________________ - Unknown (88) - Refuses to answer (99) |
| 1. Did you pay any fees at the health facility for today’s HIV care and treatment appointment?   FOR MISSED APPOINTMENT PARTICIPANTS: Did you pay any fees for your last HIV care and treatment appointment? | - Yes (1) - No (2) - Unknown (88) - Refuses to answer (99) |
| 14a. If yes to (14), what services did you pay for today/your last HIV care and treatment appointment? | - Facility user fee (1) - Medications (2) - Laboratory tests (3) - Radiology tests (4) - Other, *please specify* - Unknown (88) - Refuses to answer (99) |
| 14b. *If yes to (14)*, how much did you pay in total fees? | - ____________________________ francs - Unknown (88) - Refuses to answer (99) |
| 1. Do you pay for any medications and/or tests related to your HIV care and treatment? | - Yes, medications only (1) - Yes, tests only (2) - Yes, both medication and tests (3) - No (4) - Unknown (88) - Refuses to answer (99) |
| 15a. *If yes, medications to (15)*, how much did you pay for this medication each time you get a refill? | - ____________________________ francs - Unknown (88) - Refuses to answer (99) |
| 15b. *If yes, medications to (15)*, how often do you refill this medication? | - Once a month (1) - Every two months (2) - Every three months (3) - Every four months (4) - Every six months (5) - Once a year (6) - Other, *enter frequency* - Unknown (88) - Refuses to answer (99) |
| 15c. *If yes, tests to (15)*, how much did you pay for the test the last time it was performed? | - ____________________________ francs - Unknown (88) - Refuses to answer (99) |
| 15d. *If yes, tests to (15)*, how often do you pay for this test to be performed? | - Once a month (1) - Every two months (2) - Every three months (3) - Every four months (4) - Every six months (5) - Once a year (6) - Other, enter frequency __________ - Unknown (88) - Refuses to answer (99) |
| 15e. *If yes, medications to (15)*, have you skipped any refills for this medication over the past 12 months? | - Yes (1) - No (2) - Unknown (88) - Refuses to answer (99) |
| 15f. *If yes to (15e)*, for what reason or reasons did you skip refills over the last twelve months?  *Interviewer to read list – Choose all that apply* | - Forgot (1) - Too expensive/could not afford (2) - Did not want to take the medicine (3) - Other, *please specify* - Unknown (88) - Refuses to answer (99) |
| 15g. *If the participant provided multiple reasons to (15f)*, of these reasons, what is the primary reason you skipped refill(s) over the past 12 months? | - Forgot (1) - Too expensive/could not afford (2) - Did not want to take the medicine (3) - Other, *please specify* - Refuses to answer (99) |
| 1. Do you pay for other ancillary costs (other than user fees, medications, and tests) related to your current appointment or HIV care and treatment? | - Yes (1) - No (2) - Unknown (88) - Refuses to answer (99) |
| 16a. *If yes to (16)*, how much did you pay for the ancillary costs? | - ____________________________ francs - Unknown (88) - Refuses to answer (99) |
| 16b. *If yes to (16)*, what was the reason(s) for these ancillary costs | - _____________________________ francs |
| 16c. *If yes to (16)*, how often do you pay for these ancillary costs? | - Once a month (1) - Every two months (2) - Every three months (3) - Every four months (4) - Every six months (5) - Once a year (6) - Other, enter frequency __________ - Unknown (88) - refuses to answer (99) |
| 1. In general, what is the main mode of travel that you use to come to the facility for HIV care? | - Public transport (1) - Personal transport (2) - Walking (3) - Other, *please specify* - Refuses to answer (99) |
| 17a. how much do you pay for the one way trip here (just for yourself, not for anyone who came with you)? | - ____________________________ francs - Refuses to answer (99) |
| 17b. In general, do you pay for one person to come with you for your HIV care and treatment appointment at the facility? | - Yes (1) - No (2) - Unknown/Refuses to answer (99) |
| 1. In general, do you lose any wages or earnings (money earned) to attend your appointment? | - Yes (1) - No (2) - Refuses to answer (99) |
| 18a. *If yes to (18)*, approximately how much do you lose? | - ____________________________ francs - Unknown (88) - Refuses to answer (99) |
| 1. Do you leave your children with anyone to attend your appointment? | - Yes (1) - No (2) - N/A (No children) (3) - Refuses to answer (99) |
| 19a. *If yes to (19)*, do you pay them money or compensate them in another way, e.g., with food or other goods or services? | - Do not pay them (1) - Pay them with money (2) - Other compensation, *please specify* - Refuses to answer (99) |
| 19b. *If money*, how much money? | - ____________________________ francs - Unknown/Refuses to answer (99) |
| 1. Over the past 12 months, have you been admitted overnight to stay at a health facility or hospital due to HIV-related complications or illness? | - Yes (1) - No (2) - Refuses to answer (99) |
| 20a. *If yes to (20)*, how many times over the past 12 months have you been admitted overnight to stay at a health facility or hospital due to HIV-related complications or illness? | - _______________________________________ - Unknown (88) - Refuses to answer (99) |
| 20b. *If yes to (20)*, how much money was spent on treatment and services  received during the most recent overnight stay? We want to know about all the  costs for the stay, including any charges for laboratory tests, drugs, or other items. | - ____________________________ francs - Unknown (88) - Refuses to answer (99) |
| 1. How many scheduled HIV care and treatment appointments have you missed over the past 12 months?   FOR MISSED APPOINTMENT PARTICIPANTS: Please include your most recent missed appointment in this total. | - _______________________________________ - Unknown (88) - Refuses to answer (99) |
| 21a. For what reason or reasons did you miss scheduled HIV care and treatment appointment(s) over the past 12 months?  *Interviewer to read list*  *Multiple choices answer* | - Forgot (1) - Too expensive/could not afford (2) - Could not leave work/school/family (3) - Travelling/away from home (4) - Ill/unable to travel because of symptoms (5) - Did not appreciate the service offered during the last visit prior to the missed appointment(s) (6) - Side effects of ART (7) - Other, *please specify* - Refuses to answer (99) |
| 21b. *If the participant provided multiple reasons to (20b)*, of these reasons, what is the primary reason you missed scheduled HIV care and treatment appointment(s) over the past 12 months? | - Forgot (1) - Too expensive/could not afford (2) - Could not leave work/school/family (3) - Travelling/away from home (4) - Ill/unable to travel because of symptoms (5) - Did not appreciate the service offered during the last visit prior to the missed appointment(s) (6) - Side effects of ART (7) - Other, *please specify* - Refuses to answer (99) |
| 21c. *If the participant provided the primary reason to (21b)*, do you think that this reason could lead you to stop HIV care and treatment? | - Yes (1) - No (2) - Unknown (88) - Refuses to answer (99) |
| 1. Over the past 12 months, have you been told by a doctor or other health worker that you have high blood pressure or hypertension? | - Yes (1) - No (2) - Unknown (88) - Refuses to answer (99) |
| 22a. *If yes to (22)*, has a doctor or other health care worker prescribed medication to control your blood pressure? | - Yes (1) - No (2) - Unknown (88) - Refuses to answer (99) |
| 22b. *If yes to (22a)*, are you currently taking medication to control your blood pressure? | - Yes (1) - No (2) - Unknown (88) - Refuses to answer (99) |
| 22c. *If yes to (22b)*, how much did you pay for this medication each time you get a refill? | - ____________________________ francs - Unknown (88) - Refuses to answer (99) |
| 22d. *If yes to (22b)*, how often do you refill this medication? | - Once a month (1) - Every two months (2) - Every three months (3) - Every four months (4) - Every six months (5) - Once a year (6) - Other, enter frequency __________ - Unknown (88) - Refuses to answer (99) |
| 1. In the past 12 months, have you been told by a doctor or other health worker that you have high blood sugar or diabetes? | - Yes (1) - No (2) - Unknown (88) - Refuses to answer (99) |
| 23a. *If yes to (23)*, has a doctor or other health care worker prescribed medication to control your blood sugar? | - Yes (1) - No (2) - Unknown (88) - Refuses to answer (99) |
| 23b. *If yes to (23a)*, are you currently taking medication to control your blood sugar? | - Yes (1) - No (2) - Unknown (88) - Refuses to answer (99) |
| 23c. *If yes to (23b)*, how much did you pay for this medication each time you get a refill? | - ____________________________ francs - Unknown/Refuses to answer (99) |
| 23d. *If yes to (23b)*, how often do you refill this medication? | - Once a month (1) - Every two months (2) - Every three months (3) - Every four months (4) - Every six months (5) - Once a year (6) - Other, enter frequency __________ - Unknown (88) - Refuses to answer (99) |
| 1. Have you been told by a doctor or other health worker that you have heart disease or a chronic heart condition? | - Yes (1) - No (2) - Unknown (88) - Refuses to answer (99) |
| 24a. *If yes to (*24*)*, over the past 12 months, have you received any treatment for heart disease or chronic heart condition? | - Yes (1) - No (2) - Unknown (88) - Refuses to answer (99) |
| 24b. *If yes to (*24*a)*, how much did you pay for this treatment over the past 12 months? *Please include the facility user fee and any expenses for other items including drugs and tests,* and *please exclude any costs related to overnight stays at a health facility*? | - ____________________________ francs - Unknown (88) - Refuses to answer (99) |
| 1. Have you been told by a doctor or other health worker that you have lung disease or a chronic lung condition? | - Yes (1) - No (2) - Unknown (88) - Refuses to answer (99) |
| 25a. *If yes to (25)*, over the past 12 months, have you received any treatment for lung disease or chronic lung condition? | - Yes (1) - No (2) - Unknown (88) - Refuses to answer (99) |
| 25b. *If yes to (25a)*, how much did you pay for this treatment over the past 12 months? *Please include the facility user fee and any expenses for other items including drugs and tests,* and *please exclude any costs related to overnight stays at a health facility*? | - ____________________________ francs - Unknown (88) - Refuses to answer (99) |
| 1. Have you been told by a doctor or other health worker that you have cancer or a tumor? | - Yes (1) - No (2) - Unknown (88) - Refuses to answer (99) |
| 26a. *If yes to (26)*, over the past 12 months, have you received any treatment for cancer or a tumor? | - Yes (1) - No (2) - Unknown (88) - Refuses to answer (99) |
| 26b. *If yes to (26a)*, how much did you pay for this treatment over the past 12 months? *Please include the facility user fee and any expenses for other items including drugs and tests,* and *please exclude any costs related to overnight stays at a health facility*? | - ____________________________ francs - Unknown (88) - Refuses to answer (99) |
| 1. Have you been told by a doctor or other health worker that you have depression? | - Yes (1) - No (2) - Unknown (88) - Refuses to answer (99) |
| 27a. *If yes to (27)*, over the past 12 months, have you received any treatment for depression? | - Yes (1) - No (2) - Unknown (88) - Refuses to answer (99) |
| 27b. *If yes to (27a)*, how much did you pay for this treatment over the past 12 months? *Please include the facility user fee and any expenses for other items including drugs and tests,* and *please exclude any costs related to overnight stays at a health facility*? | - ____________________________ francs - Unknown (88) - Refuses to answer (99) |
| 1. Have you been told by a doctor or other health worker that you have any other chronic disease or disease that is long lasting? | - Yes, *specify chronic disease* (1) - No (2) - Unknown (88) - Refuses to answer (99) |
| 28a. *If yes to (28)*, are you currently taking any medication or receiving treatment for this disease? | - Yes, medication (1) - Yes, other treatment (2) - No (3) - Unknown (88) - Refuses to answer (99) |
| 28b. *If yes, medication to (28a)*, how much did you pay for this medication each time you get a refill? | - ____________________________ francs - Unknown (88) - Refuses to answer (99) |
| 28c. *If yes, medication to (28a)*, how often do you refill this medication? | - Once a month (1) - Every two months (2) - Every three months (3) - Every four months (4) - Every six months (5) - Once a year (6) - Other, *enter frequency* - Unknown (88) - Refuses to answer (99) |
| 28d. *If yes, treatment to (28a)*, over the past 12 months, have you received any treatment for this disease? | - Yes (1) - No (2) - Unknown (88) - Refuses to answer (99) |
| 28e. *If yes, treatment to (28a)*, how much did you pay for this treatment over the past 12 months? *Please include the facility user fee and any expenses for other items including drugs and tests,* and *please exclude any costs related to overnight stays at a health facility.* | - ____________________________ francs - Unknown (88) - Refuses to answer (99) |
| 1. *If yes to being prescribed medications or treatment for any of the chronic conditions (22-28)*, have you ever skipped a refill or treatment for your chronic disease over the past 12 months? | - Yes (1) - No (2) - Refuses to answer (99) |
| 29a. *If yes to (29)*, for what reason or reasons did you skip refill(s) or treatment over the last 12 months?  *Interviewer to read list*  *Multiple choices answer* | - Forgot (1) - Too expensive/could not afford (2) - Could not leave work/school/family (3) - Travelling/away from home (4) - Ill/unable to travel because of symptoms (5) - Did not appreciate the service offered during the last visit prior to the missed appointment(s) (6) - Side effects of ART (7) - Did not want treatment/to take the medicine (8) - Other, *please specify* - Refuses to answer (99) |
| 29b. *If the participant provided multiple reasons to (29a)*, of these reasons, what is the primary reason you skipped refill(s) or treatment over the past 12 months? | - Forgot (1) - Too expensive/could not afford (2) - Could not leave work/school/family (3) - Travelling/away from home (4) - Ill/unable to travel because of symptoms (5) - Did not appreciate the service offered during the last visit prior to the missed appointment(s) (6) - Side effects of ART (7) - Did not want treatment/to take the medicine (8) - Other, *please specify*: - Refuses to answer (99) |
| 1. *If yes to any of the chronic conditions (22-28)*, do you receive care and treatment of this/these condition(s) during your HIV care treatment appointments? | - Yes, high blood pressure or hypertension (1) - Yes, high blood sugar or diabetes (2) - Yes, heart disease or chronic heart condition (3) - Yes, lung disease or chronic lung condition (4) - Yes, cancer or tumor (5) - Yes, depression (6) - Yes, other chronic disease or disease that is long lasting (7) - No (8) - Refuses to answer |
| 1. *If yes to any of the chronic conditions (22-28)*, how often have you had scheduled appointments at a health facility for care and treatment of this/these condition(s) *in addition to your HIV care and treatment appointments* over the past 12 months? *Note: This should not include scheduled overnight stays at hospitals for treatment.* | - Once a month (1) - Every two months (2) - Every three months (3) - Every four months (4) - Every six months (5) - Once a year (6) - Other, *enter frequency* - Unknown (88) - Refuses to answer (99) |
| 31a. When appointments for chronic disease care do not coincide with those for HIV care, do you pay a facility user fee for these appointments? | - Yes (1) - No (2) - Unknown (88) - Refuses to answer (99) |
| 31b. *If yes to (31a)*, how much do you pay? *Please provide your best estimate of the average facility user fee.* | - ____________________________ francs - Unknown (88) - Refuses to answer (99) |
| 1. *If yes to any of the chronic conditions (22-28)*, over the past 12 months, have you missed any scheduled appointments at the facility for the care and treatment of any chronic diseases only? (e.g., not appointments for HIV or for combined HIV/NCD management) | - Yes (1) - No (2) - Unknown - Refuses to answer (99) |
| *32a. If yes to (32)*, how many scheduled appointments at the facility for the care and treatment of chronic diseases (only) have you missed over the past 12 months? | - ____________________________ - Unknown (88) - Refuses to answer (99) |
| 32b. *If yes to (32)*, for what reason or reasons did you miss scheduled appointments for the care and treatment of chronic diseases over the past 12 months?  *Interviewer to read list*  *Multiple choices answer* | - Forgot (1) - Too expensive/could not afford (2) - Could not leave work/school/family (3) - Travelling/away from home (4) - Ill/unable to travel because of symptoms (5) - Did not appreciate the service offered during the last visit prior to the missed appointment(s) (7) - Side effects of ART (8) - Other, *please specify* - Unknown (88) - Refuses to answer (99) |
| 32c. *If the participant provided multiple reasons to (32b)*, of these reasons, what is the primary reason you missed scheduled appointments for the care and treatment of chronic diseases over the past 12 months? | - Forgot (1) - Too expensive/could not afford (2) - Could not leave work/school/family (3) - Travelling/away from home (4) - Ill/unable to travel because of symptoms (5) - Did not appreciate the service offered during the last visit prior to the missed appointment(s) (6) - Side effects of ART (7) - Other, *please specify* - Refuses to answer (99) |
| 1. *If yes to any of the chronic conditions (22-28)*, do you pay to travel to the facility for these scheduled appointments only for chronic disease? | - Yes (1) - No (2) - Refuses to answer (99) |
| 33a. *If yes to (33)*, how much do you pay for a one way trip (just for yourself, not for anyone who came with you)? | - ____________________________ francs - Unknown (88) - Refuses to answer (99) |
| 1. *If yes to any of the chronic conditions (22-28)*, do you lose any wages or earnings (money earned) to attend these appointments at the facility only for chronic disease care? | - Yes (1) - No (0) - Unknown (88) - Refuses to answer (99) |
| 34a. *If yes to (34)*, how much do you lose? | - ____________________________ francs - Unknown (88) - Refuses to answer (99) |
| 1. *If yes to any of the chronic conditions (22-28)*, do you leave your children with anyone to attend these appointments at the facility only for chronic disease care? | - Yes (1) - No (2) - Not applicable (No children) (3) - Refuses to answer (99) |
| 35a. *If yes to (35)*, do you pay them with money or compensate them in a different way (e.g., with food or other goods or services?)? | - Do not pay them (1) - Pay them with money (2) - Other compensation, *please specify (3)* - Refuses to answer (99) |
| 35b. *If money*, how much money? | - ____________________________ francs - Refuses to answer (99) |
| 1. *If yes to any of the chronic conditions (22-28),* over the past 12 months, have you been admitted overnight to stay at a health facility or hospital due to chronic condition(s)? | - Yes (1) - No (2) - Refuses to answer (99) |
| 36a. *If yes to (36)*, how many times over the past 12 months have you been admitted overnight to stay at a health facility or hospital due to a chronic condition(s)? | - _______________________________________ - Unknown (88) - Refuses to answer (99) |
| 36b. *If yes to (36)*, how much money was spent on treatment and services  received during the most recent overnight stay? We want to know about all the  costs for the stay, including any charges for laboratory tests, drugs, or other items. | - ____________________________ francs - Unknown (88) - Refuses to answer (99) |
| 1. Are any other members of your household receiving HIV care and treatment? | - Yes (1) - No (2) - Unknown (88) - Refuses to answer (99) |
| 37a. *If yes to (37),* how many other members of your household are receiving HIV care and treatment? | - _______________________________________ - Unknown (88) - Refuses to answer (99) |
| 1. Are any other members of your household taking medication or receiving treatment for a chronic or long lasting disease? | - Yes (1) - No (2) - Unknown (88) - Refuses to answer (99) |
| 38a. *If yes to (38),* how many other members of your household are taking medication or receiving treatment for a chronic or long lasting disease? | - _______________________________________ - Unknown (88) - Refuses to answer (99) |
| 1. To pay for health care related costs, have you or any member of your household borrowed money since you started ART? | - Yes, myself (1) - Yes, a member of my household (2) - No (3) - Unknown (88) - Refuses to answer (99) |
| 1. To pay for health care related costs, have you or any member of your household sold property, belongings, or other assets since you started ART? | - Yes, myself (1) - Yes, a member of my household (2) - No (2) - Unknown (88) - Refuses to answer (99) |
| 1. Since you started ART, have you or any member of your household used savings to pay for health care related costs? | - Yes, myself (1) - Yes, a member of my household (2) - No (3) - Unknown (88) - Refuses to answer (99) |
