# Supplementary material for: Financial burdens of HIV and chronic disease on people living with HIV in Côte d’Ivoire: A cross-sectional out-of-pocket expenditure study
Source: PLoS One. 2021 Jul 29;16(7):e0255074. doi: 10.1371/journal.pone.0255074 (PMC8320983; doi:10.1371/journal.pone.0255074)
Supplement: S2 File — (DOCX) [file pone.0255074.s002.docx]

| 1. Code de l’établissement OpCon   *À compléter par l’enquêteur* |  |
| --- | --- |
| 1. Numéro du/de la participant(e) OpCon   *À compléter par l’enquêteur* |  |
| 1. Comment le/la participant(e) est-il interrogé(e) ?   *À compléter par l’enquêteur* | - En personne dans l’établissement (1) - Par téléphone (2) |
| 1. Quel est le sexe du/de la participant(e) ?   *À compléter par l’enquêteur* | - Homme (1) - Femme (2) - Inconnu (99) |
| 1. Quel âge aviez-vous à votre dernier anniversaire ? | - _______________________________________ [ans] - Ne sait pas/ne souhaite pas répondre (99) |
| 1. Quel est le plus haut niveau de scolarité que vous ayez atteint ? | - Aucun (1) - Primaire (2) - Secondaire (3) - Supérieur (4) - Ne souhaite pas répondre (99) |
| 1. Quel est votre statut matrimonial ? | - Marié(e) ou en concubinage (1) - Divorcé(e)/séparé(e) (2) - Veuf/ve (3) - N'a jamais été marié(e) ni vécu en concubinage (4) - Ne souhaite pas répondre (99) |
| 1. Quelle est votre occupation principale, autrement dit, quel type de travail faites-vous principalement ?   *L’enquêteur lit la liste* | - Agriculteur (1) - Professionnel du secteur public (2) - Professionnel du secteur privé (3) - Chef d’entreprise/activité libérale (commerçant, par exemple) (4) - Autre activité (travaille dans un magasin, par exemple) (5) - Services (femme de ménage, jardinier, par exemple) (6) - Étudiant(e) (7) - Travailleur/euse du sexe (8) - Aucune (9) - Autre, *veuillez préciser*(10) - Ne souhaite pas répondre (99) |
| 1. Quel a été le montant de vos revenus le mois dernier ?   *Veuillez tenir compte des revenus issus de votre travail (paie, salaire, pourboires, etc.), les revenus d’investissements, les pensions, et les versements d’allocations publiques ou de l’assistance sociale. Ne prenez pas en considération les rentrées d’argent ponctuelles, telles qu’un héritage ou une indemnité d’assurance, ni la valeur des marchandises produites destinées au troc ou à votre consommation personnelle.* | - < 22 393 francs (1) - Entre 22 394 et 32 340 francs (2) - Entre 32 341 et 60 000 francs(3) - Entre 60 001 et 120 000 francs (4) - Entre 120 001 et 300 000 francs (5) - Entre 300 001 et 600 000 francs (6) - > 600 000 francs (7) - Ne sait pas (88) - Ne souhaite pas répondre (99) |
| 1. Combien de personnes vivent actuellement dans votre ménage ? | - _______________________________________ - Ne sait pas (88) - Ne souhaite pas répondre (99) |
| 10a. Combien de personnes âgées de plus de 18 ans vivent actuellement dans votre ménage ? | - _______________________________________ - Ne sait pas (88) - Ne souhaite pas répondre (99) |
| 1. Quel a été le montant (global) des revenus de votre ménage pour les 12 derniers mois ?   *Veuillez fournir la meilleure estimation possible.*  *Veuillez tenir compte des revenus issus de votre travail (paie, salaire, pourboires, etc.), les revenus d’investissements, les pensions, et les versements d’allocations publiques ou de l’assistance sociale. Ne prenez pas en considération les rentrées d’argent ponctuelles, telles qu’un héritage ou une indemnité d’assurance, ni la valeur des marchandises produites destinées au troc ou à votre consommation personnelle.* | - < 268 716 francs (1) - Entre 268 717 et 388 080 francs (2) - Entre 388 081 et 720 000 francs (3) - Entre 720 001 et 1 440 000 francs (4) - Entre 1 440 001 et 3 600 000 francs (5) - Entre 3 600 001 et 7 200 000 francs (6) - > 7 200 00 francs (7) - Ne sait pas (88) - Ne souhaite pas répondre (99) |
| 1. Avez-vous une assurance maladie ? | - Oui, l’assurance maladie nationale (1) - Oui, l’assurance maladie communautaire (2) - Oui, une assurance privée (3) - Non (4) - Ne sait pas (88) - Ne souhaite pas répondre (99) |
| 1. Combien de fois vous êtes-vous rendu(e) dans un établissement de santé pour des rendez-vous liés au traitement et aux soins du VIH au cours des 12 derniers mois ? *Veuillez inclure la consultation d'aujourd'hui dans le total.*   POUR LES PARTICIPANT(E)S AYANT MANQUÉ LEUR RENDEZ-VOUS : Combien de fois vous êtes-vous rendu(e) à (*nom de l’établissement*) pour des rendez-vous liés au traitement et aux soins du VIH au cours des 12 derniers mois ? | - _______________________________________ - Ne sait pas (88) - Ne souhaite pas répondre (99) |
| 1. Avez-vous payé des frais à l'établissement de santé pour votre rendez-vous d'aujourd'hui lié au traitement et aux soins du VIH ?   POUR LES PARTICIPANT(E)S AYANT MANQUÉ LEUR RENDEZ-VOUS : Avez-vous payé des frais à l'établissement de santé pour votre dernier rendez-vous lié au traitement et aux soins du VIH ? | - Oui (1) - Non (2) - Ne sait pas (88) - Ne souhaite pas répondre (99) |
| 14a. *Si la réponse à la question (14) est « Oui »*, quels services avez-vous dus payer aujourd'hui/lors de votre dernier rendez-vous pour le traitement et les soins du VIH ? | - Frais de consultation (1) - Médicaments (2) - Analyses de laboratoire (3) - Examens radiologiques (4) - Autre, *veuillez préciser :* - Ne sait pas (88) - Ne souhaite pas répondre (99) |
| 14b. *Si la réponse à la question (14) est « Oui »*, quel a été le montant total de ces frais ? | - ____________________________ francs - Ne sait pas (88) - Ne souhaite pas répondre (99) |
| 1. Payez-vous certains médicaments et/ou examens/analyses liés à votre traitement et à vos soins du VIH ? | - Oui, les médicaments uniquement (1) - Oui, les examens/analyses uniquement (2) - Oui, les médicaments et les examens/analyses - Non (4) - Ne sait pas (88) - Ne souhaite pas répondre (99) |
| 15a. *Si la réponse à la question (15) est « Oui, les médicaments »*, combien vous coûte ce médicament chaque fois que vous renouvelez votre ordonnance ? | - ____________________________ francs - Ne sait pas (88) - Ne souhaite pas répondre (99) |
| 15b. *Si la réponse à la question (15) est « Oui, les médicaments »*, à quelle fréquence renouvelez-vous l’ordonnance pour ce médicament ? | - Une fois par mois (1) - Tous les deux mois (2) - Tous les trois mois (3) - Tous les quatre mois (4) - Tous les six mois (5) - Une fois par an (6) - Autre, *veuillez préciser la fréquence* - Ne sait pas (88) - Ne souhaite pas répondre (99) |
| 15c. *Si la réponse à la question (15) est « Oui, les examens/analyses »*, combien vous a coûté l’examen/l'analyse la dernière fois qu'il/elle a été réalisé(e) ? | - ____________________________ francs - Ne sait pas (88) - Ne souhaite pas répondre (99) |
| 15d. *Si la réponse à la question (15) est « Oui, les examens/analyses »*, à quelle fréquence payez-vous pour réaliser cet examen/cette analyse ? | - Une fois par mois (1) - Tous les deux mois (2) - Tous les trois mois (3) - Tous les quatre mois (4) - Tous les six mois (5) - Une fois par an (6) - Autre, veuillez préciser la fréquence __________ - Ne sait pas (88) - Ne souhaite pas répondre (99) |
| 15e. *Si la réponse à la question (15) est « Oui, les médicaments »*, avez-vous manqué un ou plusieurs renouvellements pour ce médicament au cours des 12 derniers mois ? | - Oui (1) - Non (2) - Ne sait pas (88) - Ne souhaite pas répondre (99) |
| 15f. *Si la réponse à la question (15e) est « Oui »*, pour quelle(s) raison(s) avez-vous manqué des renouvellements au cours des 12 derniers mois ?  *L’enquêteur lit la liste – Choisissez toutes les réponses applicables* | - J'ai oublié (1) - Les médicaments étaient trop chers/je ne pouvais pas me le permettre (2) - Je ne voulais pas prendre le médicament (3) - Autre, *veuillez préciser :* - Ne sait pas (88) - Ne souhaite pas répondre (99) |
| 15g. *Si le/la participant(e) a indiqué plusieurs raisons à la question (15f)*, parmi ces raisons, quelle est la raison principale pour laquelle vous avez manqué un (ou plusieurs) renouvellement(s) au cours des 12 derniers mois ? | - J'ai oublié (1) - Les médicaments étaient trop chers/je ne pouvais pas me le permettre (2) - Je ne voulais pas prendre le médicament (3) - Autre, *veuillez préciser :* - Ne souhaite pas répondre (99) |
| 1. Avez-vous dû payer des frais annexes (autres que les frais de consultation, les médicaments et les examens/analyses) liés à votre rendez-vous d’aujourd’hui ou à vos soins et votre traitement actuels du VIH ? | - Oui (1) - Non (2) - Ne sait pas (88) - Ne souhaite pas répondre (99) |
| 16a. *Si la réponse à la question (16) est « oui »,* combien ces frais annexes vous ont-ils coûté ? | - ____________________________ francs - Ne sait pas (88) - Ne souhaite pas répondre (99) |
| 16b. *Si la réponse à la question (16) est « oui »,* quelle(s) étai(ent) la/les raison(s) de ces frais annexes ? | - _____________________________ francs |
| 16c. *Si la réponse à la question (16) est « oui »,* à quelle fréquence devez-vous payer ces frais annexes ? | - Une fois par mois (1) - Tous les deux mois (2) - Tous les trois mois (3) - Tous les quatre mois (4) - Tous les six mois (5) - Une fois par an (6) - Autre, veuillez préciser : __________ - Ne sait pas (88) - Ne souhaite pas répondre (99) |
| 1. En général, quel moyen de déplacement principal utilisez-vous pour vous rendre à l’établissement en vue de recevoir vos soins liés au VIH ? | - Moyen de transport public (1) - Moyen de transport personnel (2) - Marche à pied (3) - Autre, *veuillez préciser* - Ne souhaite pas répondre (99) |
| 17a. Combien vous a coûté le trajet aller pour venir ici (uniquement pour vous, pas pour les personnes qui vous accompagnent) ? | - ____________________________ francs - Ne souhaite pas répondre (99) |
| 17b. En règle générale, engagez-vous également des frais pour qu’une personne vous accompagne à l’établissement de santé lors de vos rendez-vous pour le traitement et les soins du VIH ? | - Oui (1) - Non (2) - Ne sait pas/ne souhaite pas répondre (99) |
| 1. En général, le fait de venir à vos rendez-vous vous fait-il perdre une partie de votre salaire ou de vos revenus (argent que vous gagnez) ? | - Oui (1) - Non (2) - Ne souhaite pas répondre (99) |
| 18a. *Si la réponse à la question (18) est « Oui »*, combien cela vous fait-il perdre d'argent environ ? | - ____________________________ francs - Ne sait pas (88) - Ne souhaite pas répondre (99) |
| 1. Confiez-vous vos enfants à quelqu’un pour venir à votre rendez-vous ? | - Oui (1) - Non (2) - S/O (pas d’enfant) (3) - Ne souhaite pas répondre (99) |
| 19a. *Si la réponse à la question (19) est « Oui »*, versez-vous de l’argent à cette personne ou la dédommagez-vous d'une autre façon, en lui offrant, en échange, de la nourriture, des biens ou des services ? | - Je ne la paie pas (1) - Je lui donne de l’argent (2) - Je la dédommage autrement, *veuillez préciser* - Ne souhaite pas répondre (99) |
| 19b. *Si le/la participant(e) lui verse de l’argent*, quelle somme lui versez-vous ? | - ____________________________ francs - Ne sait pas/ne souhaite pas répondre (99) |
| 1. Au cours des 12 derniers mois, vous est-il arrivé de devoir passer la nuit dans un établissement de santé ou à l’hôpital à cause de complications ou d'une maladie liées au VIH ? | - Oui (1) - Non (2) - Ne souhaite pas répondre (99) |
| 20a. *Si la réponse à la question (20) est « Oui »*, combien de fois au cours des 12 derniers mois avez-vous dû passer la nuit dans un établissement de santé ou à l’hôpital à cause de complications ou d'une maladie liées au VIH ? | - _______________________________________ - Ne sait pas (88) - Ne souhaite pas répondre (99) |
| 20b. *Si la réponse à la question (20) est « Oui »*, combien ont coûté le traitement et les services reçus lors de votre dernière hospitalisation dans un établissement de santé ? Nous aimerions connaître tous les frais liés au séjour, y compris pour les analyses de laboratoire, les médicaments et autres. | - ____________________________ francs - Ne sait pas (88) - Ne souhaite pas répondre (99) |
| 1. Combien de rendez-vous de suivi programmés pour le traitement et les soins du VIH avez-vous manqués au cours des 12 derniers mois ?   POUR LES PARTICIPANT(E)S AYANT MANQUÉ LEUR RENDEZ-VOUS : *Veuillez inclure le dernier rendez-vous manqué dans ce total.* | - _______________________________________ - Ne sait pas (88) - Ne souhaite pas répondre (99) |
| 21a. Pour quelle(s) raison(s) avez-vous manqué un (ou plusieurs) rendez-vous de suivi programmé(s) pour le traitement et les soins du VIH au cours des 12 derniers mois ?  *L’enquêteur lit la liste*  *Plusieurs réponses possibles* | - J'ai oublié (1) - Les médicaments étaient trop chers/je ne pouvais pas me le permettre (2) - Je ne pouvais pas manquer le travail/l’école/laisser ma famille (3) - J'étais en voyage/je n’étais pas chez moi (4) - J'étais malade/je n’étais pas en mesure de me déplacer à cause de mes symptômes (5) - Je n’ai pas apprécié les services offerts lors de la dernière visite précédent le(s) rendez-vous manqué(s) (6) - À cause des effets indésirables du TAR (7) - Autre, *veuillez préciser :* - Ne souhaite pas répondre (99) |
| 21b. *Si le/la participant(e) a indiqué plusieurs raisons à la question (20b)*, parmi ces raisons, quelle est la raison principale pour laquelle vous avez manqué un (ou plusieurs) rendez-vous de suivi programmé(s) pour le traitement et les soins du VIH au cours des 12 derniers mois ? | - J'ai oublié (1) - Les médicaments étaient trop chers/je ne pouvais pas me le permettre (2) - Je ne pouvais pas manquer le travail/l’école/laisser ma famille (3) - J'étais en voyage/je n’étais pas chez moi (4) - J'étais malade/je n’étais pas en mesure de me déplacer à cause de mes symptômes (5) - Je n’ai pas apprécié les services offerts lors de la dernière visite précédent le(s) rendez-vous manqué(s) (6) - À cause des effets indésirables du TAR (7) - Autre, *veuillez préciser :* - Ne souhaite pas répondre (99) |

| 21c. *Si le/la participant(e) a indiqué la raison principale à la question (21b)*, pensez-vous que cette raison aurait pu vous conduire à arrêter votre traitement et vos soins du VIH ? | - Oui (1) - Non (2) - Ne sait pas (88) - Ne souhaite pas répondre (99) |
| --- | --- |

| 1. Au cours des 12 derniers mois, un médecin ou un autre agent de santé vous a-t-il dit que votre tension artérielle est élevée ou que vous souffrez d'hypertension ? | - Oui (1) - Non (2) - Ne sait pas (88) - Ne souhaite pas répondre (99) |
| --- | --- |
| 22a. *Si la réponse à la question (22) est « Oui »*, un médecin ou un autre agent de santé vous a-t-il prescrit des médicaments pour contrôler votre tension artérielle ? | - Oui (1) - Non (2) - Ne sait pas (88) - Ne souhaite pas répondre (99) |
| 22b. *Si la réponse à la question (22a) est « Oui »*, prenez-vous des médicaments pour contrôler votre tension artérielle ? | - Oui (1) - Non (2) - Ne sait pas (88) - Ne souhaite pas répondre (99) |
| 22c. *Si la réponse à la question (22b) est « Oui »*, combien vous coûte ce médicament chaque fois que vous renouvelez votre ordonnance ? | - ____________________________ francs - Ne sait pas (88) - Ne souhaite pas répondre (99) |
| 22d. *Si la réponse à la question (22b) est « Oui »*, à quelle fréquence renouvelez-vous ce médicament ? | - Une fois par mois (1) - Tous les deux mois (2) - Tous les trois mois (3) - Tous les quatre mois (4) - Tous les six mois (5) - Une fois par an (6) - Autre, veuillez préciser la fréquence __________ - Ne sait pas (88) - Ne souhaite pas répondre (99) |
| 1. Au cours des 12 derniers mois, un médecin ou un autre agent de santé vous a-t-il dit que votre glycémie (taux de sucre dans le sang) est élevée ou que vous souffrez de diabète ? | - Oui (1) - Non (2) - Ne sait pas (88) - Ne souhaite pas répondre (99) |
| 23a. *Si la réponse à la question (23) est « Oui »*, un médecin ou un autre agent de santé vous a-t-il prescrit des médicaments pour contrôler votre glycémie ? | - Oui (1) - Non (2) - Ne sait pas (88) - Ne souhaite pas répondre (99) |
| 23b. *Si la réponse à la question (23a) est « Oui »*, prenez-vous des médicaments pour contrôler votre glycémie ? | - Oui (1) - Non (2) - Ne sait pas (88) - Ne souhaite pas répondre (99) |
| 23c. *Si la réponse à la question (23b) est « Oui »*, combien vous coûte ce médicament chaque fois que vous renouvelez votre ordonnance ? | - ____________________________ francs - Ne sait pas/ne souhaite pas répondre (99) |
| 23d. *Si la réponse à la question (23b) est « Oui »*, à quelle fréquence renouvelez-vous ce médicament ? | - Une fois par mois (1) - Tous les deux mois (2) - Tous les trois mois (3) - Tous les quatre mois (4) - Tous les six mois (5) - Une fois par an (6) - Autre, veuillez préciser la fréquence __________ - Ne sait pas (88) - Ne souhaite pas répondre (99) |
| 1. Un médecin ou un autre agent de santé vous a-t-il dit que vous êtes atteint(e) d’une maladie cardiaque ou d’insuffisance cardiaque chronique ? | - Oui (1) - Non (2) - Ne sait pas (88) - Ne souhaite pas répondre (99) |
| 24a. *Si la réponse à la question (*24*) est « Oui »*, au cours des 12 derniers mois, avez-vous reçu un traitement pour une maladie cardiaque ou une insuffisance cardiaque chronique ? | - Oui (1) - Non (2) - Ne sait pas (88) - Ne souhaite pas répondre (99) |
| 24b. *Si la réponse à la question (24a) est « Oui »*, combien ce traitement vous a-t-il coûté au cours des 12 derniers mois ? *Veuillez inclure les frais de consultation et toute autre dépense liée à d'autres éléments tels que les médicaments et les analyses/examens* et *ne pas tenir compte des coûts liés à une hospitalisation dans l'établissement de santé.* | - ____________________________ francs - Ne sait pas (88) - Ne souhaite pas répondre (99) |
| 1. Un médecin ou un autre agent de santé vous a-t-il dit que vous êtes atteint(e) d’une maladie pulmonaire ou d'une affection pulmonaire chronique ? | - Oui (1) - Non (2) - Ne sait pas (88) - Ne souhaite pas répondre (99) |
| 25a. *Si la réponse à la question (*25*) est « Oui »*, au cours des 12 derniers mois, avez-vous reçu un traitement pour une maladie pulmonaire ou une affection pulmonaire chronique ? | - Oui (1) - Non (2) - Ne sait pas (88) - Ne souhaite pas répondre (99) |
| 25b. *Si la réponse à la question (25) est « Oui »*, combien ce traitement vous a-t-il coûté au cours des 12 derniers mois ? *Veuillez inclure les frais de consultation et toute autre dépense liée à d'autres éléments tels que les médicaments et les analyses/examens* et *ne pas tenir compte des coûts liés à un séjour dans l'établissement de santé.* | - ____________________________ francs - Ne sait pas (88) - Ne souhaite pas répondre (99) |
| 1. Un médecin ou un autre agent de santé vous a-t-il dit que vous êtes atteint(e) d'un cancer ou d'une tumeur ? | - Oui (1) - Non (2) - Ne sait pas (88) - Ne souhaite pas répondre (99) |
| 26a. *Si la réponse à la question (26) est « Oui »*, au cours des 12 derniers mois, avez-vous reçu un traitement pour un cancer ou une tumeur ? | - Oui (1) - Non (2) - Ne sait pas (88) - Ne souhaite pas répondre (99) |
| 26b. *Si la réponse à la question (26a) est « Oui »*, combien ce traitement vous a-t-il coûté au cours des 12 derniers mois ? *Veuillez inclure les frais de consultation et toute autre dépense liée à d'autres éléments tels que les médicaments et les analyses/examens* et *ne pas tenir compte des coûts liés à un séjour dans l'établissement de santé.* | - ____________________________ francs - Ne sait pas (88) - Ne souhaite pas répondre (99) |
| 1. Un médecin ou un autre agent de santé vous a-t-il dit que vous êtes atteint(e) de dépression ? | - Oui (1) - Non (2) - Ne sait pas (88) - Ne souhaite pas répondre (99) |
| 27a. *Si la réponse à la question (27) est « Oui »*, au cours des 12 derniers mois, avez-vous reçu un traitement pour la dépression ? | - Oui (1) - Non (2) - Ne sait pas (88) - Ne souhaite pas répondre (99) |
| 27b. *Si la réponse à la question (27a) est « Oui »*, combien ce traitement vous a-t-il coûté au cours des 12 derniers mois ? *Veuillez inclure les frais de consultation et toute autre dépense liée à d'autres éléments tels que les médicaments et les analyses/examens* et *ne pas tenir compte des coûts liés à un séjour dans l'établissement de santé.* | - ____________________________ francs - Ne sait pas (88) - Ne souhaite pas répondre (99) |
| 1. Un médecin ou un autre agent de santé vous a-t-il dit que vous êtes atteint(e) d’une autre maladie chronique ou affection de longue durée ? | - Oui, *veuillez préciser la maladie chronique* (1) - Non (2) - Ne sait pas (88) - Ne souhaite pas répondre (99) |
| 28a. *Si la réponse à la question (28) est « Oui »*, prenez-vous actuellement des médicaments ou recevez-vous un traitement pour cette maladie ? | - Oui, des médicaments (1) - Oui, un autre traitement (2) - Non (3) - Ne sait pas (88) - Ne souhaite pas répondre (99) |
| 28b. *Si la réponse à la question (28a) est « Oui »*, combien vous coûte ce médicament chaque fois que vous renouvelez votre ordonnance ? | - ____________________________ francs - Ne sait pas (88) - Ne souhaite pas répondre (99) |
| 28c. *Si la réponse à la question (28a) est « Oui, des médicaments »*, à quelle fréquence renouvelez-vous l’ordonnance pour ce médicament ? | - Une fois par mois (1) - Tous les deux mois (2) - Tous les trois mois (3) - Tous les quatre mois (4) - Tous les six mois (5) - Une fois par an (6) - Autre, *veuillez préciser la fréquence* - Ne sait pas (88) - Ne souhaite pas répondre (99) |
| 28d. *Si la réponse à la question (28a) est « Oui »*, au cours des 12 derniers mois, avez-vous reçu un traitement pour cette maladie ? | - Oui (1) - Non (2) - Ne sait pas (88) - Ne souhaite pas répondre (99) |
| 28e. *Si la réponse à la question (28a) est « Oui »*, combien ce traitement vous a-t-il coûté au cours des 12 derniers mois ? *Veuillez inclure les frais de consultation et toute autre dépense liée à d'autres éléments tels que les médicaments et les analyses/examens* et *ne pas tenir compte des coûts liés à un séjour dans l'établissement de santé.* | - ____________________________ francs - Ne sait pas (88) - Ne souhaite pas répondre (99) |
| 1. *Si des médicaments ou un traitement ont été prescrits pour l’une de ces maladies chroniques (réponse « Oui » aux questions 22-28)*, vous est-il arrivé de manquer un renouvellement d'ordonnance ou un traitement pour votre maladie chronique au cours des 12 derniers mois ? | - Oui (1) - Non (2) - Ne souhaite pas répondre (99) |
| 29a. *Si la réponse à la question (29) est « Oui »*, pour quelle(s) raison(s) avez-vous manqué un (ou plusieurs) renouvellement(s) ou traitement(s) au cours des 12 derniers mois ?  *L’enquêteur lit la liste*  *Plusieurs réponses possibles* | - J'ai oublié (1) - Les médicaments étaient trop chers/je ne pouvais pas me le permettre (2) - Je ne pouvais pas manquer le travail/l’école/laisser ma famille (3) - J'étais en voyage/je n’étais pas chez moi (4) - J'étais malade/je n’étais pas en mesure de me déplacer à cause de mes symptômes (5) - Je n’ai pas apprécié les services offerts lors de la dernière visite précédent le(s) rendez-vous manqué(s) (6) - À cause des effets indésirables du TAR (7) - Je ne voulais pas de traitement/prendre le médicament (8) - Autre, *veuillez préciser :* - Ne souhaite pas répondre (99) |
| 29b. *Si le/la participant(e) a indiqué plusieurs raisons à la question (29a)*, parmi ces raisons, quelle est la raison principale pour laquelle vous avez manqué un (ou plusieurs) renouvellement(s) ou traitement(s) au cours des 12 derniers mois ? | - J'ai oublié (1) - Les médicaments étaient trop chers/je ne pouvais pas me le permettre (2) - Je ne pouvais pas manquer le travail/l’école/laisser ma famille (3) - J'étais en voyage/je n’étais pas chez moi (4) - J'étais malade/je n’étais pas en mesure de me déplacer à cause de mes symptômes (5) - Je n’ai pas apprécié les services offerts lors de la dernière visite précédent le(s) rendez-vous manqué(s) (6) - À cause des effets indésirables du TAR (7) - Je ne voulais pas de traitement/prendre le médicament (8) - Autre, *veuillez préciser :* - Ne souhaite pas répondre (99) |
| 1. *Si la réponse à l’une des questions sur les maladies chroniques (22-28) est « Oui »*, avez-vous reçu des soins et un traitement pour cette(ces) maladie(s) pendant vos rendez-vous de suivi pour le traitement et les soins du VIH ? | - Oui, pour une tension artérielle élevée ou une hypertension (1) - Oui, pour une glycémie élevée ou un diabète (2) - Oui, pour une maladie cardiaque ou une insuffisance cardiaque chronique (3) - Oui, pour une maladie pulmonaire ou une affection pulmonaire chronique (4) - Oui, pour un cancer ou une tumeur (5) - Oui, pour une dépression (6) - Oui, pour une autre maladie chronique ou affection de longue durée (7) - Non (8) - Ne souhaite pas répondre |
| 1. *Si la réponse à l’une des questions sur les maladies chroniques (22-28) est « Oui »*, à quelle fréquence avez-vous eu des rendez-vous programmés à l'établissement de santé pour les soins et traitements liés à cette/ces affection(s) en plus des rendez-vous de suivi pour le traitement et les soins du VIH au cours des 12 derniers mois ? *Remarque : la réponse ne doit pas inclure les hospitalisations programmées pour le traitement.* | - Une fois par mois (1) - Tous les deux mois (2) - Tous les trois mois (3) - Tous les quatre mois (4) - Tous les six mois (5) - Une fois par an (6) - Autre, *veuillez préciser la fréquence* - Ne sait pas (88) - Ne souhaite pas répondre (99) |
| 31a. Lorsque les rendez-vous de soins pour votre maladie chronique ne coïncident pas avec les rendez-vous de soins du VIH, payez-vous des frais de consultation pour ces rendez-vous ? | - Oui (1) - Non (2) - Ne sait pas (88) - Ne souhaite pas répondre (99) |
| 31b. *Si la réponse à la question (31a) est « Oui »*, combien cela vous coûte-t-il ? *Veuillez indiquer votre meilleure estimation du montant moyen des frais de consultation.* | - ____________________________ francs - Ne sait pas (88) - Ne souhaite pas répondre (99) |
| 1. *Si la réponse à l’une des questions sur les maladies chroniques (22-28) est « Oui »*, au cours des 12 derniers mois, avez-vous manqué un ou plusieurs rendez-vous programmé(s) à l’établissement de soins pour le traitement et les soins d'une maladie chronique uniquement ? (à savoir : pas les rendez-vous pour la prise en charge du VIH ou la prise en charge combinée du VIH et de la MNT) | - Oui (1) - Non (2) - Ne sait pas (88) - Ne souhaite pas répondre (99) |
| *32a. Si la réponse à la question (32) est « Oui »*, combien de rendez-vous de suivi programmés à l’établissement de soins pour le traitement et les soins d’une maladie chronique (uniquement) avez-vous manqués au cours des 12 derniers mois ? | - ____________________________ - Ne sait pas (88) - Ne souhaite pas répondre (99) |
| 32b. *Si la réponse à la question (32) est « Oui »*, pour quelle(s) raison(s) avez-vous manqué un (ou plusieurs) rendez-vous de suivi programmé(s) pour le traitement et les soins d'une maladie chronique au cours des 12 derniers mois ?  *L’enquêteur lit la liste*  *Plusieurs réponses possibles* | - J'ai oublié (1) - Les médicaments ou le traitement étaient trop chers/je ne pouvais pas me le permettre (2) - Je ne pouvais pas manquer le travail/l’école/laisser ma famille (3) - J'étais en voyage/je n’étais pas chez moi (4) - J'étais malade/je n’étais pas en mesure de me déplacer à cause de mes symptômes (5) - Je n’ai pas apprécié les services offerts lors de la dernière visite précédent le(s) rendez-vous manqué(s) (6) - À cause des effets indésirables du TAR (7) - Autre, *veuillez préciser :* - Ne sait pas (88) - Ne souhaite pas répondre (99) |
| 32c. *Si le/la participant(e) a indiqué plusieurs raisons à la question (32b)*, parmi ces raisons, quelle est la raison principale pour laquelle vous avez manqué un (ou plusieurs) rendez-vous de suivi programmé(s) pour le traitement et les soins d'une maladie chronique au cours des 12 derniers mois ? | - J'ai oublié (1) - Les médicaments ou le traitement étaient trop chers/je ne pouvais pas me le permettre (2) - Je ne pouvais pas manquer le travail/l’école/laisser ma famille (3) - J'étais en voyage/je n’étais pas chez moi (4) - J'étais malade/je n’étais pas en mesure de me déplacer à cause de mes symptômes (5) - Je n’ai pas apprécié les services offerts lors de la dernière visite précédent le(s) rendez-vous manqué(s) (6) - À cause des effets indésirables du TAR (7) - Autre, *veuillez préciser :* - Ne souhaite pas répondre (99) |
| 1. *Si la réponse à l’une des questions sur les maladies chroniques (22-28) est « Oui »*, payez-vous le trajet jusqu'à l’établissement pour ces rendez-vous de suivi programmés uniquement pour votre maladie chronique ? | - Oui (1) - Non (2) - Ne souhaite pas répondre (99) |
| 33a. *Si la réponse à la question (33) est « Oui »*, combien vous coûte le trajet aller (uniquement pour vous, pas pour les personnes qui vous accompagnent) ? | - ____________________________ francs - Ne sait pas (88) - Ne souhaite pas répondre (99) |
| 1. *Si la réponse à l’une des questions sur les maladies chroniques (22-28) est « Oui »*, le fait de vous rendre à ces rendez-vous à l’établissement de santé uniquement pour les soins liés à votre maladie chronique vous fait-il perdre une partie de votre salaire ou de vos revenus (argent que vous gagnez) ? | - Oui (1) - Non (2) - Ne sait pas (88) - Ne souhaite pas répondre (99) |
| 34a. *Si la réponse à la question (34) est « Oui »*, combien cela vous fait-il perdre d'argent ? | - ____________________________ francs - Ne sait pas (88) - Ne souhaite pas répondre (99) |
| 1. *Si la réponse à l’une des questions sur les maladies chroniques (22-28) est « Oui »*, confiez-vous vos enfants à quelqu’un lorsque vous allez à ces rendez-vous à l’établissement de santé uniquement pour les soins liés à votre maladie chronique ? | - Oui (1) - Non (2) - S/O (pas d’enfant) (3) - Ne souhaite pas répondre (99) |
| 35a. *Si la réponse à la question (35) est « Oui »*, versez-vous de l’argent à cette personne ou la dédommagez-vous d'une autre façon (en lui offrant, en échange, de la nourriture, des biens ou des services) ? | - Je ne la paie pas (1) - Je lui donne de l’argent (2) - Je la dédommage autrement, *veuillez préciser (3)* - Ne souhaite pas répondre (99) |
| 35b. *Si le/la participant(e) lui verse de l’argent*, quelle somme lui versez-vous ? | - ____________________________ francs - Ne souhaite pas répondre (99) |
| 1. *Si la réponse à l’une des questions sur les maladies chroniques (22-28) est « Oui »*, au cours des 12 derniers mois, avez-vous dû passer la nuit dans un établissement de santé ou à l’hôpital à cause d'une maladie chronique ? | - Oui (1) - Non (2) - Ne souhaite pas répondre (99) |
| 36a. *Si la réponse à la question (36) est « Oui »*, combien de fois au cours des 12 derniers mois avez-vous dû passer la nuit dans un établissement de santé ou à l’hôpital pour une maladie chronique ? | - _______________________________________ - Ne sait pas (88) - Ne souhaite pas répondre (99) |
| 36b. *Si la réponse à la question (36) est « Oui »*, combien ont coûté le traitement et les services reçus lors de votre dernière hospitalisation dans un établissement de santé ? Nous aimerions connaître tous les frais liés au séjour, y compris pour les analyses de laboratoire, les médicaments et autres. | - ____________________________ francs - Ne sait pas (88) - Ne souhaite pas répondre (99) |
| 1. D'autres membres de votre ménage reçoivent-ils un traitement et des soins pour le VIH ? | - Oui (1) - Non (2) - Ne sait pas (88) - Ne souhaite pas répondre (99) |
| 37a. *Si la réponse à la question (37) est « Oui »,* combien d'autres personnes au sein de votre ménage reçoivent un traitement et des soins pour le VIH ? | - _______________________________________ - Ne sait pas (88) - Ne souhaite pas répondre (99) |
| 1. D'autres membres de votre ménage prennent-ils des médicaments ou reçoivent-ils un traitement pour une maladie chronique ou une affection de longue durée ? | - Oui (1) - Non (2) - Ne sait pas (88) - Ne souhaite pas répondre (99) |
| 38a. *Si la réponse à la question (38) est « Oui »,* combien d'autres personnes au sein de votre ménage reçoivent des médicaments ou un traitement pour une maladie chronique ou une affection de longue durée ? | - _______________________________________ - Ne sait pas (88) - Ne souhaite pas répondre (99) |
| 1. Pour payer les frais liés à vos soins de santé, avez-vous dû ou un membre de votre ménage a-t-il dû emprunter de l’argent depuis que vous avez commencé votre TAR ? | - Oui, moi (1) - Oui, un membre de mon ménage (2) - Non (3) - Ne sait pas (88) - Ne souhaite pas répondre (99) |
| 1. Pour payer les frais liés à vos soins de santé, avez-vous dû ou un membre de votre ménage a-t-il dû vendre des terres, des biens ou d'autres actifs depuis que vous avez commencé votre TAR ? | - Oui, moi (1) - Oui, un membre de mon ménage (2) - Non (3) - Ne sait pas (88) - Ne souhaite pas répondre (99) |
| 1. Depuis que vous avez commencé votre TAR, avez-vous ou un membre de votre ménage a-t-il puisé dans vos/ses économies pour payer les frais liés aux soins de santé ? | - Oui, moi (1) - Oui, un membre de mon ménage (2) - Non (3) - Ne sait pas (88) - Ne souhaite pas répondre (99) |
